# Mechanisms of competitive selection: A canonical neural circuit framework

**DOI:** 10.7554/eLife.51473

**Published:** 2020-05-20

**Authors:** Shreesh P Mysore, Ninad B Kothari

**Affiliations:** 1 Department of Psychological and Brain Sciences, Johns Hopkins UniversityBaltimoreUnited States; 2 The Solomon H. Snyder Department of Neuroscience, Johns Hopkins UniversityBaltimoreUnited States; University of PennsylvaniaUnited States; University of PennsylvaniaUnited States

**Keywords:** selection, selective attention, perceptual decision-making, value-based choice, circuit, computation

## Abstract

Competitive selection, the transformation of multiple competing sensory inputs and internal states into a unitary choice, is a fundamental component of animal behavior. Selection behaviors have been studied under several intersecting umbrellas including decision-making, action selection, perceptual categorization, and attentional selection. Neural correlates of these behaviors and computational models have been investigated extensively. However, specific, identifiable neural circuit mechanisms underlying the implementation of selection remain elusive. Here, we employ a first principles approach to map competitive selection explicitly onto neural circuit elements. We decompose selection into six computational primitives, identify demands that their execution places on neural circuit design, and propose a canonical neural circuit framework. The resulting framework has several links to neural literature, indicating its biological feasibility, and has several common elements with prominent computational models, suggesting its generality. We propose that this framework can help catalyze experimental discovery of the neural circuit underpinnings of competitive selection.

## Introduction

Animals inhabit complex environments in which they routinely encounter multiple competing options. At every moment, they must select the most relevant or beneficial one, and execute the appropriate behavior. At an elemental level, selection among options to approach or retreat from a stimulus, and to fight or take flight, can impact the very survival of the animal. More generally, selection is an integral aspect of nearly all complex cognitive functions as well as everyday behaviors. Given the critical importance of the selection of one option among many available ones to adaptive behavior, how such competitive selection is implemented in the brain is a fundamental question in behavioral neuroscience.

In the literature, competitive selection has been considered in a variety of contexts including perceptual categorization, action choice, decision-making and attention. For instance, central to selective attention is the problem of identifying the next target among multiple stimuli to guide behavior (Knudsen, 2007; Moore and Zirnsak, 2017; but see also McMains and Somers, 2004; Cavanagh and Alvarez, 2005). Selecting the location of the most salient or the most behaviorally relevant stimulus while ignoring distractors (Bichot and Schall, 1999; Bichot et al., 2001; Ikeda and Hikosaka, 2003; McPeek and Keller, 2004), and responding to image components that are related to a specific feature but not others (O'Craven et al., 1999; Mante et al., 2013) are just a few examples from the literature on selective attention. Central to value-based decision-making is the challenge of choosing the most beneficial option among competing alternatives (Padoa-Schioppa, 2011). The choice between a small but immediate reward versus a large but delayed reward, and between sure rewards versus risky (probabilistic) ones with a higher expected value, are two examples of value-based decisions that have been investigated (Roesch and Olson, 2005; Padoa-Schioppa and Assad, 2006; Kable and Glimcher, 2007; Hunt et al., 2012; Strait et al., 2014). Central to perceptual categorization is the problem of discriminating among competing percepts in the face of potentially ambiguous or noisy sensory stimuli (Hanks and Summerfield, 2017). The identification of the dominant odor component in a mixture of two (or more) odors (Niessing and Friedrich, 2010), determination of the animal category from an ambiguous visual stimulus (Freedman et al., 2001; Swaminathan and Freedman, 2012), identification of motion direction in a random dot motion task (Gold and Shadlen, 2007; Liu and Wang, 2008), and identification of a song note based on its duration (Prather et al., 2008) are just a few examples from the rich literature on the study of perceptual selection. Lastly, central to action selection is the issue of executing the appropriate motor plan among competing alternatives (Cisek and Kalaska, 2010; Murakami and Mainen, 2015). The choice between rolling or turning behaviors in fly larvae (Jovanic et al., 2016), between freezing versus flight defensive behaviors in mice in response to a threatening stimulus (Shang et al., 2015; Fadok et al., 2017; Shang et al., 2018), between grooming and freezing behaviors in mice (Hong et al., 2014), between swimming to the left versus right in zebrafish (Koyama et al., 2016), between feeding versus escape in mollusks (Kovac and Davis, 1980; Kristan, 2008), and between two reaching actions in monkeys (Cisek and Kalaska, 2005) are a few of the many examples studied in the literature.

**Figure 1.**
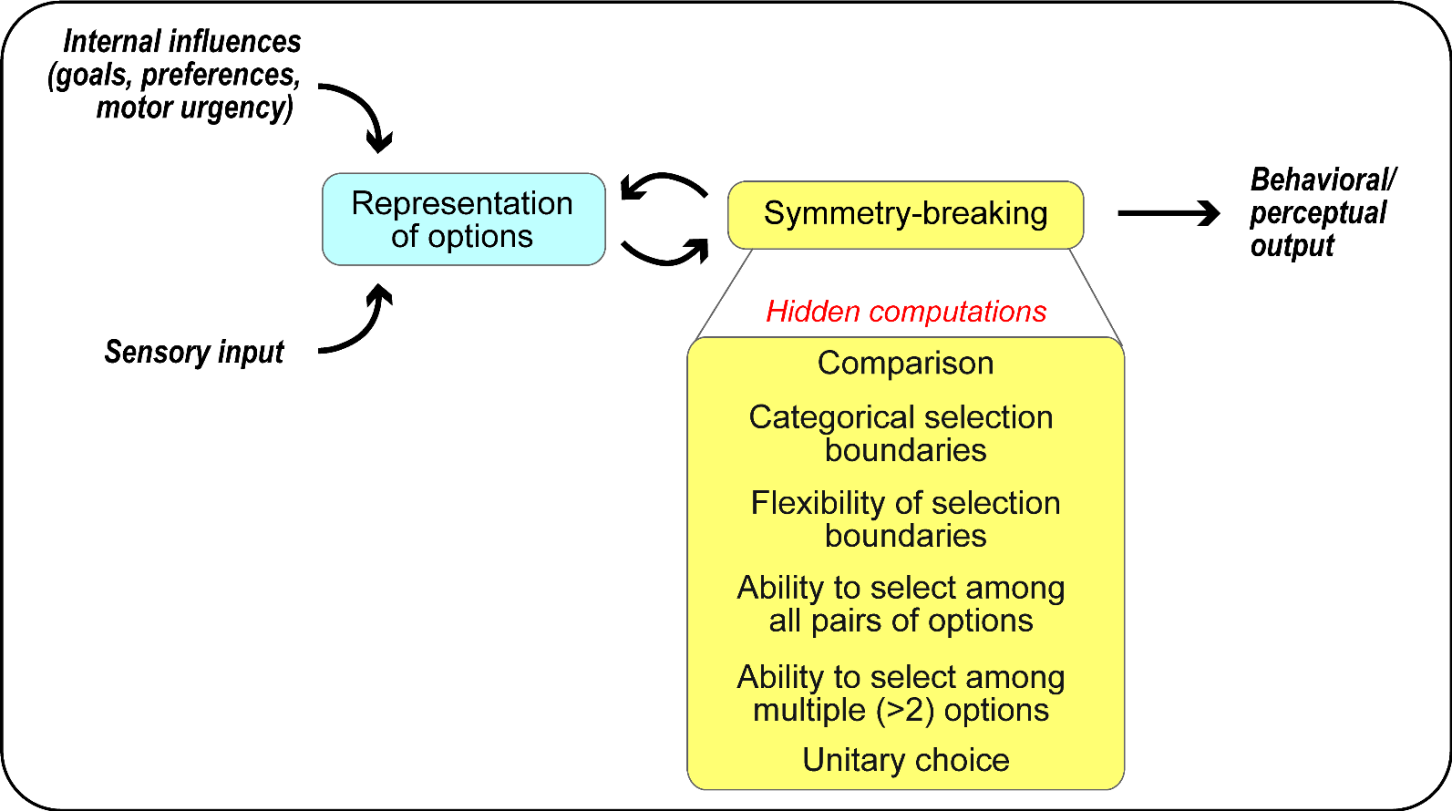
A computational framework for competitive selection based on first principles. The computational primitives underlying competitive selection are shown as ‘hidden computations’. See also Box 1.

Studies of the different forms of competitive selection across animal species have advanced our understanding of many aspects of the neural basis of selection. Prominent among these are the identities of brain areas that are involved in particular selection tasks, the information encoded by these areas in service of selection, and models of how these neural representations may contribute to observed choice behaviors. An emerging view is that despite differences in selection tasks and the brain areas studied, different forms of competitive selection may share key computational principles as well as neural mechanisms (Wang, 2008; Cisek and Kalaska, 2010; Freedman and Assad, 2011; Padoa-Schioppa, 2011; Mysore and Knudsen, 2012). However, several fundamental questions remain unanswered: (i) ‘Exactly what key computations constitute competitive selection?’, (ii) ‘What aspects of the models correspond to which computational goals?’, and more importantly, (iii) ‘How is competitive selection actually implemented within the neural circuits of the relevant brain areas?’ Here, by decomposing selection into a set of well-defined computational building blocks and mapping them onto neural circuit building blocks, we develop a neural circuit framework that can help experimenters answer these questions and elucidate neural circuit mechanisms of competitive selection (Figure 1).

### Organization

We begin with two key theoretical (and computational) perspectives on selection that have been discussed in the literature, namely the winner-take-all (WTA) and the accumulation-to-threshold (A2T) perspectives, and describe their isomorphic relationship (section titled ‘Competitive selection as symmetry-breaking: Theory and models’). Next, we break down the seemingly monolithic winner-take-all selection into six key computational primitives (section titled ‘Hidden computational features…’). Here, for each computational feature, we describe its conceptual role in the idealized WTA equation, and discuss its applicability to selection problems that have been studied in behavioral neuroscience. We then explore the demands that its execution places on neural circuit architecture and function, and identify specific circuit motif(s) that can implement the computational feature. In this process, we view the literature on selection through the lens of the elemental computation and summarize relevant findings from different species and forms of selection. Because our focus, here, is on questions of biological realization rather than in-principle operation, we draw selectively upon those studies in the literature that inform issues of neural implementation. Next, we combine the individual circuit motifs from the previous section into a mechanistic framework for competitive selection, and link this WTA-inspired framework back to the A2T perspective, thereby closing the loop between them (section titled ‘Feasibility, generality and limitations…’). In addition, we highlight the biological viability of this framework, and also point out that it has several key elements in common with leading models of decision making that have been proposed to account for behavioral as well as neural results in cortical and subcortical brain areas. Based on these, we argue that the proposed neural circuit framework is canonical - one that can serve as a starting point for experimentally uncovering how core computations of competitive selection are implemented by specific neural mechanisms. The final section aids such future efforts by detailing specific predictions that arise from each of the six computational building blocks, as well as approaches to test them experimentally (section titled ‘Experimentally testable predictions’). We conclude with a broad discussion of challenges in investigating the neural circuit bases of competitive selection.

### Definitions

#### Options

Stimuli or potential choices available to the animal. For instance, in the case of value-based decision-making, option A may be a stimulus associated with a reward of high magnitude but that arrives with long delay, and option B, a stimulus associated with a reward of low magnitude but short delay. In the case of selection for spatial attention, two competing options may be stimuli at two different locations, with one of them aligned with a spatial cue.

#### Competitive selection/decision-making

The process by which one option must be selected among many (>1) available options, that is, for which competition among neural representations is required to identify a winner. Examples include selection between two or more stimulus options presented simultaneously, selection between two or more stimulus options presented across time, or selection between two or more neural representations activated by a single (ambiguous) stimulus option. Competitive selection does not include situations in which just one representation is activated because only a single option is available (for instance, attending to a single target in the absence of any distracters), nor situations in which category boundaries are hardwired through learning stable representations in such a manner that no competition among representations is necessary for the choice.

#### Norm of an option

A quantity that represents the ‘worth’ of an option (or of the attribute being compared). This term captures more broadly, ideas encapsulated in specialized terms used in the literature. For instance, in the context of spatial attention, the norm would be the classic ‘stimulus priority’, and in the case of value-based decision-making, it could be the ‘subjective value’. This term is intended, ultimately, to correspond to a continuous quantity that allows for lawful comparisons along some common scale of comparison. (We note that our use of the term ‘norm’ is not intended to correspond directly to the formal mathematical definition of ‘norm’ on vector spaces.)

#### Channel

Group of neurons (excitatory and inhibitory) involved in representing an option.

## Competitive selection as symmetry-breaking: Theory and models

### Theoretical formulations

**Figure 2.**
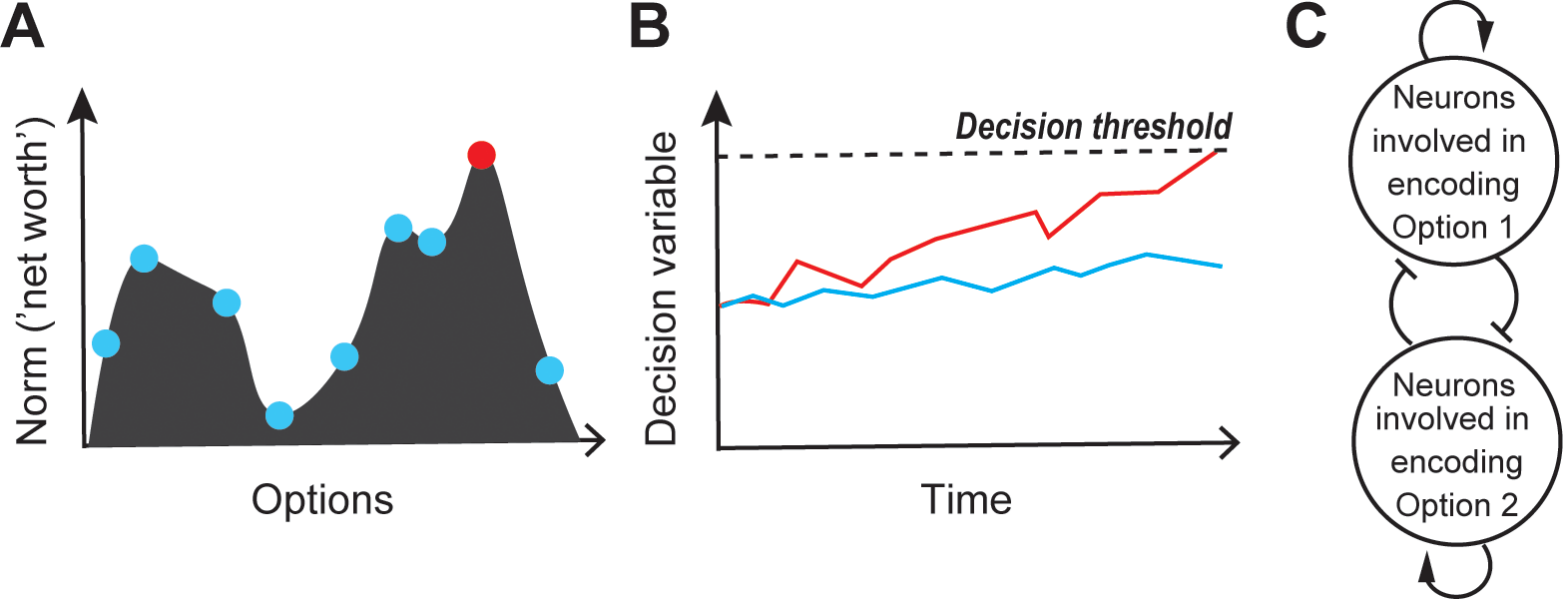
Competitive selection: theory and modeling. (**A**) Schematic illustrating the peak-detection formulation of competitive selection. Shown is a representational landscape (depicted here as a continuous ‘hilly’ surface), in which each point corresponds to an option-norm pair. Dots illustrate a few specific option-norm pairs; red dot represents the winning option, i.e., the option corresponding to the highest peak in the landscape. (**B**) Schematic illustrating the ‘first-to-threshold’ formulation. Shown is the evolution over time of two decision variables, one red and one blue. The winning option is the one (red) that first crosses decision threshold. (**C**) Modeling. A prominent network architecture involving ‘mutual inhibition’, used in both the winner-take-all (WTA) and accumulation to threshold (A2T) formulations of competitive selection (Roe et al., 2001; Usher and McClelland, 2001; Machens et al., 2005; Wong and Wang, 2006; Mysore and Knudsen, 2012; see text). Arrows with pointed heads denote excitation (recurrent), and those with flat heads denote inhibition.

Competitive selection, at its core, is a symmetry-breaking process: among several options available to the animal, one is chosen, and all the others (temporarily) rejected. From a theoretical perspective, such symmetry-breaking has been described using several equivalent formulations. It has been described as the detection of the peak in the dynamic representational landscape, in which the heights at different points represent the norms of the different options at any instant (Fecteau and Munoz, 2006; Cisek and Kalaska, 2010; Sugawara and Nikaido, 2014; Figure 2A). It has also been framed as a form of categorization - the transformation of continuous inputs (here, graded representations of the options) into two discrete output groups separated by a selection or category boundary such that the neural representation of just one of the options passes into the ‘selected’ category, whereas those of the remaining options fall into a second ‘unselected’ one (Freedman and Assad, 2011; Mysore and Knudsen, 2011b; Mysore and Knudsen, 2012). Cast in the language of dynamical systems, different choices have been described as different attractor states of a dynamic ‘energy function’ defined over the space of option attributes, with competitive selection involving the identification of the attractor with the lowest energy given sensory inputs and internal influences at that instant (Machens et al., 2005; Niessing and Friedrich, 2010). Alternatively, selection can be thought of as the outcome of a template matching process in which either the multiple options are matched to a relevant neural template, or a single option is compared to multiple neural templates, with the one yielding the best match (or lowest error) being the selected option (Riesenhuber and Poggio, 1999); selection in sequential match-to-sample experiments is consistent with this view. Finally, another commonly used description is that competitive selection occurs when the neural representation associated with one of the competing options *first* crosses some functional response threshold (Figure 2B). Ultimately, however, no matter the description, the outcome of symmetry-breaking is that the selected option, and only that one, triggers downstream consequences that culminate in a percept or behavioral output.

### Modeling formulations

From a modeling perspective, two broad classes of models have been employed to account for symmetry-breaking in competitive selection. The first is the class of winner-take-all (**WTA**) or attractor models. Here, computational units representing different options interact with one another, with the strongest option outcompeting all the others (Carpenter and Grossberg, 1987; Yuille and Grzywacz, 1989; Hahnloser et al., 2000; Maass, 2000; Rousselet et al., 2004). If *x_i_* are inputs corresponding to *i = 1,… N* options, *y_i_* are the corresponding transformed outputs, x* denotes the winning input, and i* denotes the winning option, the generalized winner-take-all operation is represented by the equations:

(1a) $$y_{i}=\left\{ \begin{array}{l} f(x_{i});if\;x_{i}\geq x_{i}\forall j\\ 0;otherwise \end{array}\right.$$

(1b) $$i^{*}=isuchthaty_{i}=f\left(x_{i}\right)$$

(1c) $$x^{*}=x_{i^{*}}$$

In other words, the option with the dominant representation breaks symmetry and drives the output. The winner-take-all class of models has been used to account for selection both among simultaneously presented options (Koch and Ullman, 1987), as well as between options presented over time (Machens et al., 2005). This class of models accounts for the majority of the theoretical formulations of selection listed above - including peak detection, attractor dynamics, categorization, and error minimizing template matching (Riesenhuber and Poggio, 1999; Machens et al., 2005; Wang, 2008; Mysore and Knudsen, 2012).

The second class of models takes the accumulation-to-threshold (A2T) approach and accounts explicitly for the theoretical formulation of earliest threshold crossing. Here, a commonly used model in two-option scenarios is the diffusion model, which implements the statistically optimal sequential probability ratio test (SPRT; Barnard, 1946; Wald, 1947; Kira et al., 2015). In this model, evidence for the options from noisy or ambiguous sensory inputs is combined into a single accumulator, with the state of the accumulator increasing when inputs provide evidence for one option, and decreasing, when evidence favors the other option (Stone, 1960; Laming, 1968; Ratcliff, 1978; Ratcliff and McKoon, 2008). The evidence accumulates until it crosses a positive (or negative) ‘threshold’. Alternatively, computational units representing each of the competing options accumulate evidence for that option towards an abstract decision threshold (Figure 2B; Roe et al., 2001; Usher and McClelland, 2004; Bogacz et al., 2007; Furman and Wang, 2008; Deco et al., 2009; Ditterich, 2010; Thura et al., 2012; Xue and Liu, 2014; Hanks and Summerfield, 2017). The option for which the evidence crosses threshold first is designated the ‘winner’ or the selected option. In other words, in the A2T formulation, the temporal primacy of threshold crossing results in symmetry-breaking and triggering of the output corresponding to the selected option. Early computational descriptions, called race models, assumed that evidence for each option accumulated independently of the others (Vickers, 1970; Vickers, 1979), but gave way to later models in which accumulation processes for the different options interacted with one another in an inhibitory manner (Chen et al., 2013; Teodorescu and Usher, 2013). In addition, the accumulation of evidence has been described both as a continuous ramping process (Shadlen and Kiani, 2013; Hanks et al., 2015) and a discrete stepping process (Latimer et al., 2015), although recent work suggest that both processes account equally well for neural data (Zoltowski et al., 2019). The A2T class of models has been used to great effect in the decision-making literature (Kim and Shadlen, 1999; Shadlen and Newsome, 2001; Roitman and Shadlen, 2002; Huk and Shadlen, 2005; Gold and Shadlen, 2007; Kim and Basso, 2008; Hanks et al., 2015; Herman et al., 2018; Yartsev et al., 2018). A major feature of A2T models is that they not only account for the choice made (given the inputs), but also account well for the reaction times of the choice responses.

Although seemingly different, the WTA and A2T classes of models are not conceptually distinct (Wang, 2012; Figure 2C), but rather view selection from two complementary perspectives. A2T models focus heavily on the time-course of evolution of the neural responses that lead to the representational landscape, and handle symmetry-breaking by building into the model, the notion of threshold crossing, albeit without much detail on how the threshold might be set (Churchland and Ditterich, 2012; Huk and Meister, 2012); but see Lo and Wang, 2006; Wei et al., 2015 for multi-area proposals). By contrast, WTA models typically begin with a representational landscape without much detail on how it might be constructed, and focus on implementing symmetry-breaking. Despite the different emphases, the WTA and A2T models are largely isomorphic, with A2T models necessarily producing a categorical choice, and WTA models being compatible with accumulation of evidence over time (Wang, 2002; Wong and Wang, 2006; Wang, 2008). This correspondence is not surprising considering that, in reality, both the evolution of neural responses over time (construction of the representational landscape), as well as the application of symmetry-breaking transformations, are crucial aspects of any functional description of competitive selection. Consequently, just as the different theoretical formulations employed to describe competitive selection are conceptually equivalent, so are the two broad classes of computational models used to account for it.

### Neural support

Studies of the different forms of selection have used both the WTA and the A2T models to describe observed neural correlates, supporting their interchangeability at the conceptual level. For instance, selection for spatial attention has been viewed from the perspective of A2T (Ratcliff et al., 2007; Mysore and Knudsen, 2014; Herman et al., 2018) as well as WTA (Fecteau and Munoz, 2006; Mysore and Knudsen, 2011b; Mysore and Knudsen, 2014). Similarly, studies of decision-making have applied A2T (Gold and Shadlen, 2007) as well as WTA models (Hunt et al., 2012; Strait et al., 2014). In the context of perceptual categorization, several studies have interpreted results using A2T (Shadlen and Newsome, 2001; Roitman and Shadlen, 2002), and others using WTA models (Wang and Spelke, 2002; Machens et al., 2005; Wong and Wang, 2006). Finally, studies have examined action selection from both the A2T perspective (Öztürk, 2009; Thura and Cisek, 2014) and the WTA perspective (Cisek, 2007; Cisek and Kalaska, 2010; Koyama et al., 2016).

Thus, there is evidence in neural responses to support aspects of both the WTA as well as the A2T formulations of symmetry-breaking. However, little is known about how either class of models is actually implemented in neural circuitry. For instance, how categorical segregation of inputs is accomplished in the brain has been unclear (Freedman and Assad, 2016). Similarly, how the values of selection thresholds are determined, whether they are static or dynamic, and how they are specified in neural circuits, are all unknown (Huk and Meister, 2012), despite biologically-grounded proposals (Lo and Wang, 2006; Wei et al., 2015). Moreover, considering the range of differences in model implementation (Ditterich, 2010; O'Connell et al., 2018), it is unclear what specific aspects of these models are required for accounting for neurophysiological and behavioral data, and what are non-essential details. In the next section, we explicitly address the circuit mechanisms of selection by adopting, predominantly, the WTA perspective (Maass, 2000).

## Hidden computational features of WTA symmetry-breaking and their implementation in neural circuits

Despite the wealth of computational models used to implement WTA selection, and their extensive invocation in the selection literature to account for neural and behavioral responses, a detailed understanding of their implementation in neural circuits has remained elusive. We propose that a key impediment has been the deceptive simplicity of the WTA transformation. Encapsulated in a two-line equation (Equation 1a), this seemingly straightforward mathematical transformation is, in fact, composed of six ‘hidden’ computational features that act together to produce *idealized* WTA selection (Figure 1, Box 1). Briefly, they are: (#1) comparison, (#2) categorical (or sharp) selection boundary, (#3) dynamic flexibility of the selection or category boundary, (#4) ability to select among many (all) viable pairs of options (independent of their identities), (#5) ability to select among multiple (>2) options, and (#6) generation of a unitary output. Each of these computations can place non-trivial constraints on the nature of interactions between the representations of the competing options and on the organization of the underlying neural circuitry. Here, we start with the representation of the options and then, by examining each of the six computational features, delineating their circuit requirements, and highlighting support (if any) from the literature, we attempt to explicitly map competitive selection onto circuit elements.

Box 1. First-principles framework for competitive selection: implementation of elemental computations.

**Figure 3.**
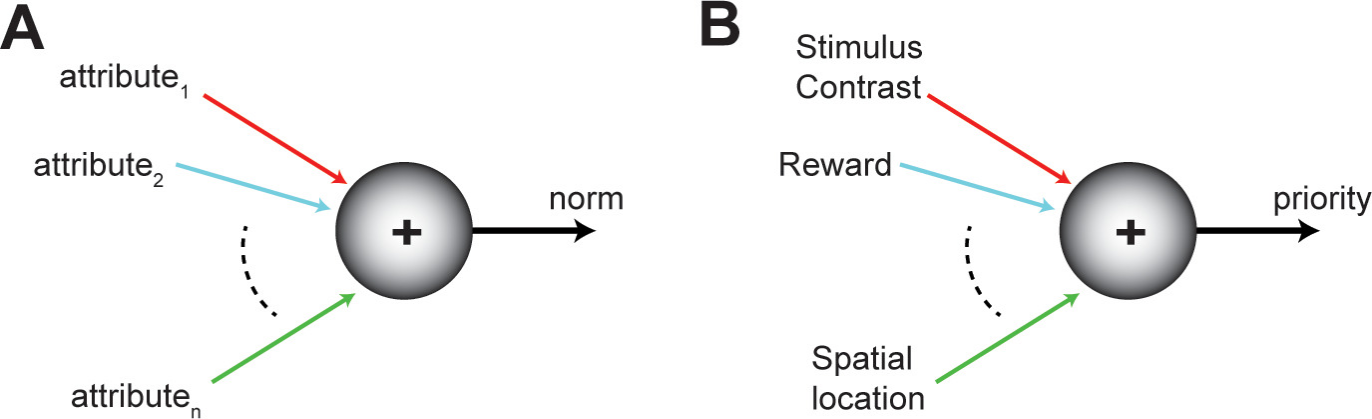
Norm of an option. (**A**) Schematic showing the norm of an option as a function of its various attributes (colored axes). The norm of an option encodes its worth to the animal, and can vary from moment to moment. The norm serves as a common frame of reference for comparing among competing options. (**B**) Illustration of the norm in the context of selective (spatial) attention.

Competitive selection is broken down into six ‘hidden’ computational features (Figure 1), by drawing inspiration from the idealized WTA equation (Equation 1 and Figure 2). These features are built on a representational foundation in which the worth of each option (or of the relevant attribute(s) of each option) to the animal, referred to broadly as the ‘norm’ of each option, is encoded in neural activity (Figure 3).

**Figure 4.**
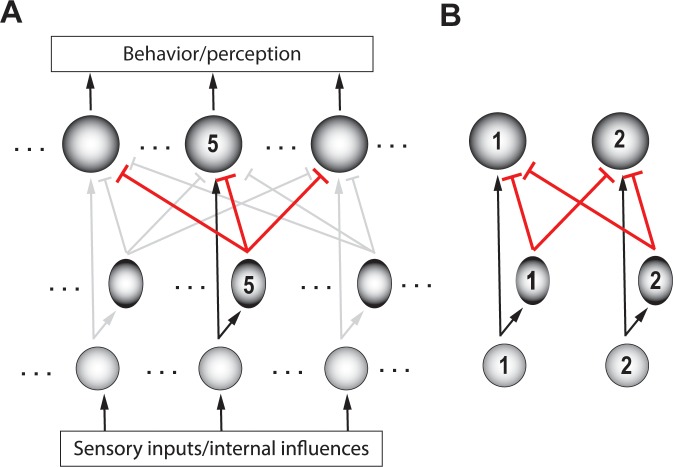
Schematic of a circuit illustrating a motif for comparison. (**A**) Rows – layers of neurons; columns – neurons encoding for different options (referred to as ‘channels’); channel #5 neurons are labeled. Bottom layer (small, grey circles) – neurons in the input layer to the selection circuit, which gather information about the various attributes of each option. Middle layer (ovals) – inhibitory neurons. Top layer (large circles) – excitatory neurons that signal the winner. Although each channel is capable of triggering output, only the winning channel will. When only a single option is presented, the corresponding output neuron signals the norm of that option. Arrows with flat heads – inhibitory projections; in black: Excitatory input corresponding to option #5; in red: global feedforward inhibition from option #5 to all options (corresponding to the first computation of comparison); in grey: Excitatory and inhibitory connections corresponding to other options. (**B**) A two-option version of the circuit in A, redrawn for clarity. Arrows depicting input into the circuit and output from the circuit are not shown here (and in subsequent figures), for simplicity.

**Figure 5.**
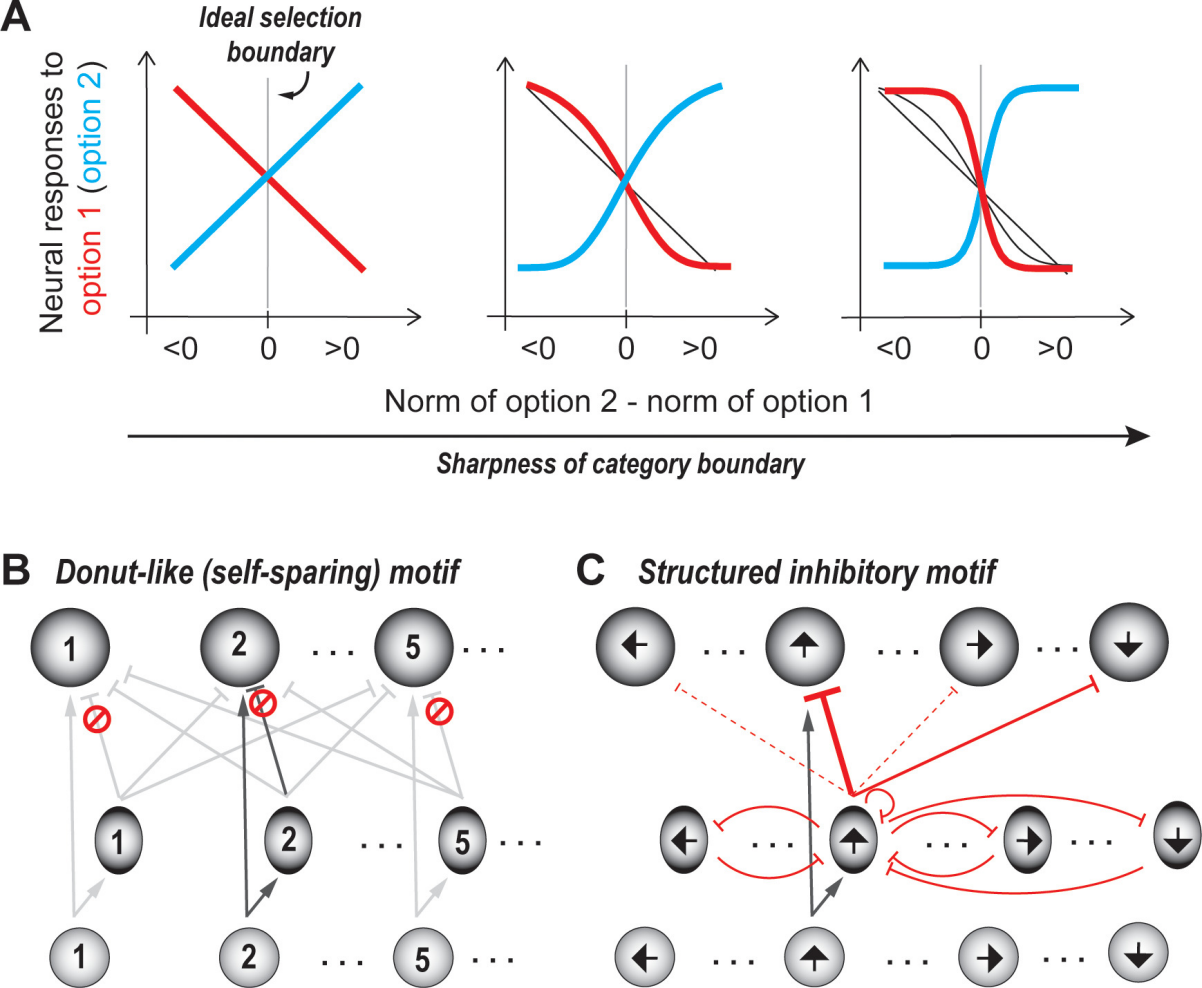
Categorical selection boundary. (**A**) Neural response curves showing different degrees of categorical selection in a two option case. X-axis represents the relative norm of the two options, i.e., the difference between the norms of option 2 and option 1. Difference >0: Option two ought to be the winner (option two is stronger than option 1);<0: Option one ought to be the winner (option two is weaker than option 1); Difference = 0/vertical gray line: ideal selection boundary. Y-axes represent the mean responses of neurons that prefer option 1 (red) and option 2 (blue). These can also be thought of as the average probability that option one is going to be signaled by these neurons as the winner. (Only the means are shown for clarity, but in reality, each point on the curve is associated with a distribution of responses.) The leftmost panel illustrates the implementation of an uncertain selection boundary, one that is most vulnerable to sensory ambiguity and neural noise; subsequent panels illustrate the implementation of increasingly categorical boundaries; based on Mahajan and Mysore, 2020. (**B**) Schematic of selection circuit illustrating the donut-like inhibitory motif for implementing categorical selection boundaries (based on Mahajan and Mysore, 2020). In this circuit, self-inhibition, both feedforward and feedback, is zero (indicated by the red symbol). All other conventions as in Figure 3. (**C**) Schematic of selection circuit illustrating the structured inhibitory motif for robust-to-noise selection in the context of a circular feature space (such as motion direction (Xue and Liu, 2014). In this circuit, feedforward inhibition to similar feature values (‘self-inhibition’) as well as to opposite feature values is strong (solid red lines), with self-inhibition being the stronger of the two (thicker red line). Feedforward inhibition to all other feature values is very weak (dashed red lines). Feedback inhibition to similar, opposite, and other feature values is of uniform strength (curved red lines).

**Figure 6.**
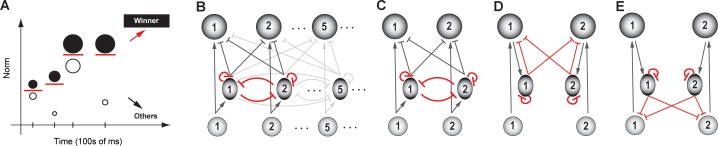
Dynamic flexibility of the selection boundary. (**A**) Illustration of the need for a dynamically flexible selection boundary. Tick marks along x-axis: individual trials, or instants at which the set of options (circles parallel to the y-axis) available to the animal changes. For illustrative purposes, we consider only two options being presented in each trial. Filled circle: option with higher norm; open circle: option with lower norm. Red horizontal line: selection boundary; must change dynamically based on the set of options to correctly signal the winner; adapted from Mysore and Knudsen, 2011a. (**B**) Schematic of selection circuit illustrating motif required for flexibility, namely, feedback inhibition among options (highlighted in red). Feedback is implemented as reciprocal inhibition of inhibition (Mysore and Knudsen, 2012). All other conventions as in Figure 4. (**C**) A two-option version of the circuit in B, redrawn for clarity. (**D, E**) Two alternatives to C for implementing feedback inhibition among the options. Both are examples of ‘indirect’ implementation, and involve more synapses and neurons within the feedback loop than the one in B/C; adapted from Mysore and Knudsen, 2012.

**Figure 7.**
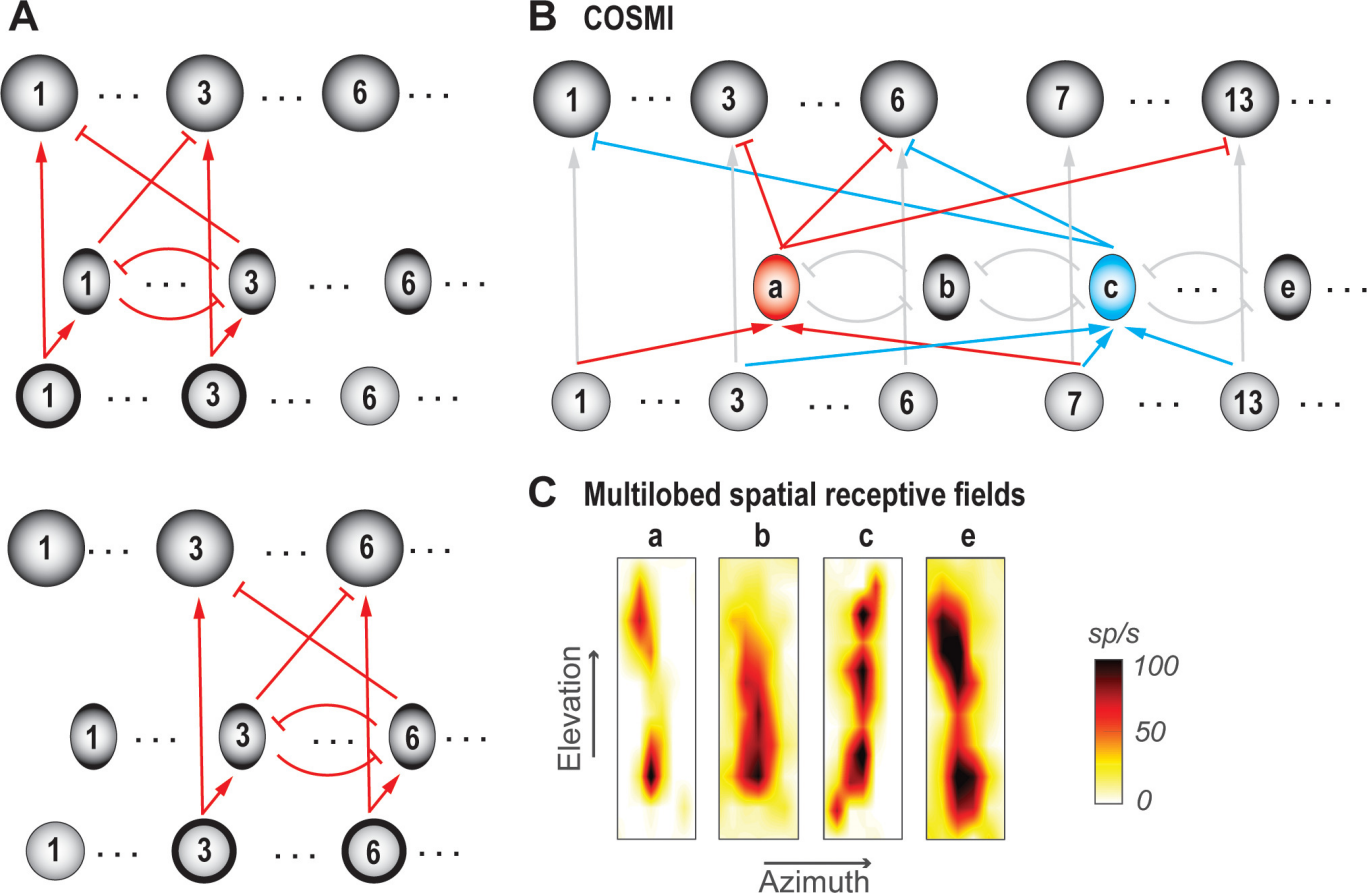
Ability to select among all viable pairs of options. (**A**) Schematics illustrating the ‘copy-and paste’ circuit strategy for achieving invariance to option identities. Top panel: Illustrates a scenario in which only options 1 and 3 are available to the animal (indicated by thick outlines around the input neurons 1 and 3 in the bottom layer). All neurons involved in encoding a particular option constitute a neural ‘channel’; shown is channel 3. Bottom panel: Illustrates a different scenario in which only options 3 and 6 are available. Here, selection between channels 3 and 6 is being solved by simply ‘copying-and-pasting’ the circuit module (red connections) used for selection between options 1 and 3. (**B**) Schematic of selection circuit illustrating the COSMI strategy for achieving invariance to option identities, discovered in the context of selection for spatial attention (Mahajan and Mysore, 2018; Mahajan and Mysore, 2020). COSMI- Combinatorially optimal coding by sparse, multilobe inhibitory neurons (see also Box 3). A group of high-firing inhibitory neurons, fewer in number than the number of spatial locations encoded (or number of channels), encode space densely with spatial receptive fields that have multiple hotspots or lobes. (**C**) Spatial receptive fields (RFs) of four Imc neurons, three of which are multilobed; neurons a,b,c,e, here, correspond loosely to the ones in B; adapted from Mahajan and Mysore, 2018.

**Figure 8.**
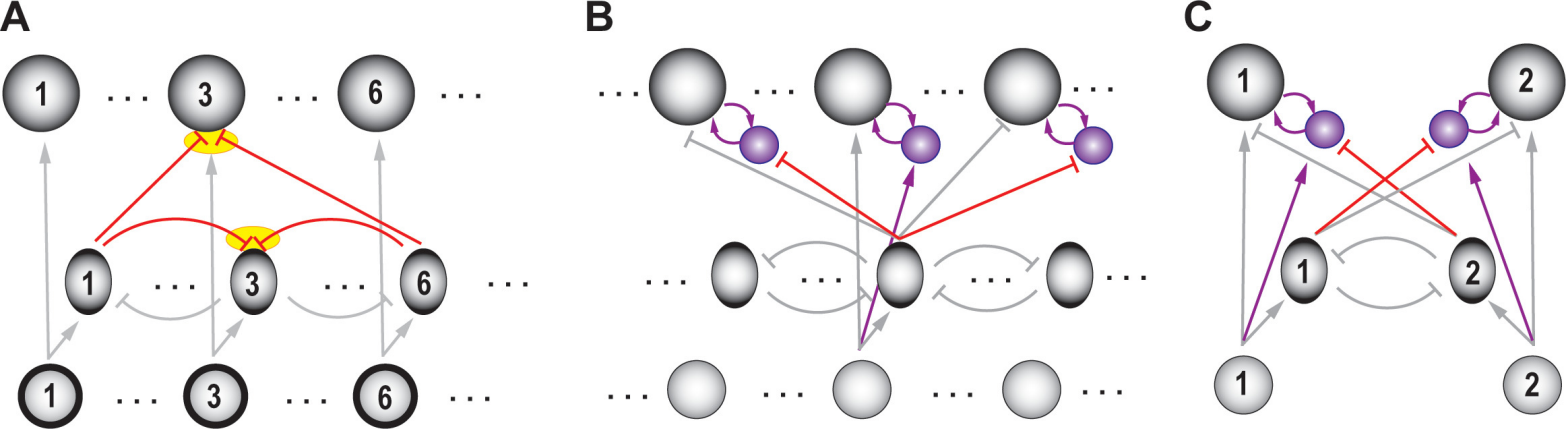
Ability to select among multiple (>2) options, and generation of unitary choice. (**A**) Ability to select among more than two options. Schematic of selection circuit illustrating a scenario in which multiple options (here, options 1, 3, and 6) are presented simultaneously. Yellow ovals: Highlights the open question pertaining to the rules by which inhibition (feedforward as well as feedback) from all other options is combined to impact the representation of each option. All other conventions as in previous figures. (**B–C**) Unitary output generation. (**B**) Schematic of selection circuit illustrating a potential circuit motif for generating unitary choice (see text). Purple circles: amplifier neurons providing recurrent excitation (purple arrows); they are also recipients of high-gain competitive inhibition in a donut-like pattern (red arrows) (Mahajan and Mysore, 2020). (**C**) A two-option version of the circuit in B, redrawn for clarity.

1. Comparison across options: This corresponds to the ‘>’ operation in the WTA equation, and can be implemented by neural inhibition among competing options, with the inhibition generated by each option being dependent on its norm (Figure 4).
2. Categorical selection boundary: The ability to select the option with the highest norm, even when competing options are similar. Within the limits posed by neural response variability, such categorization is implemented well by a donut-like pattern of competitive inhibition (Figure 5).
3. Flexibility of selection boundary: The ability to dynamically select the option with the highest norm, no matter what the actual values of the options’ norms are. This requires feedback inhibition among competing options (Figure 6).
4. Ability to select among many (all) viable pairs of competing options: This can be implemented either by copying-and-pasting the circuit module for selection among one pair of options, for all pairs of options (expensive strategy). Alternatively, it can be implemented by adopting a metabolically efficient, combinatorially optimized strategy implemented by sparse inhibitory neurons with dense coding properties (Figure 7; this strategy minimized net metabolic and wiring costs).
5. Ability to select in the presence of multiple (>2) competing options: This can be implemented by scaling up the circuit that selects between two options to multiple options, together with specific nonlinearity constraints governing the combination of inhibition from multiple sources (Figure 8A).
6. Production of unitary choice: The ability to produce exactly one winner. This can be implemented with high-gain competitive inhibition, coupled with downstream recurrent excitation (Figure 8BC).

We note that the winner-take-all equation (Equation 1a) represents the idealized implementation of selection operating with a hard (discontinuous) nonlinearity and noiseless steady-state representations. By contrast, neurons and networks typically implement softer (continuous) nonlinearities, that is, ones in which the transition between a low output state and a high output state occurs over a non-zero range of inputs. Second, neural representations possess significant trial-to-trial variability (or noise), and competitive selection transpires over time - from the instant the options are made available to the animal to the instant of expression of the behavioral or perceptual output corresponding to the chosen option. Third, although the six computations are all key parts of an idealized selection process, they may apply to varying extents to different forms of selection behavior, with the ‘comparison’ (#1) and ‘unitary choice’ (#6) features being essential across the board. Nonetheless, as we will see below, Equation (1a) and the six computational building blocks together serve as a fruitful starting point for constructing a neural circuit framework for competitive selection.

### Neural representation of options

#### Conceptual set-up

Selection operates upon the substrate of neural representations of the competing options. Therefore, a reasonable requirement for lawful comparisons among options is that their neural representations be encoded along a common scale (Levy and Glimcher, 2012), one that embodies the worth, or so-called ‘norm’, of each option (Figure 3AB). The norm can either represent the net contribution of all the attributes of an option that are relevant to the selection process, computed via some weighted combination of those of individual attributes, or can represent just the contribution of a particular attribute that may be being compared at a given instant. To be useful, the norm satisfies two properties. First, it is instantiated in neural activity via a shared or common currency (for instance, firing rate), thereby providing a level playing field for comparisons. Second, the neural encoding of the norm of an option is graded and ordered, with ‘stronger’ neural responses reflecting higher magnitudes of the norm. These properties facilitate the construction of a representational landscape in which each option evokes responses proportional to its norm (Figure 2A). We note that there is no requirement for these representations to be encoded in a separate ‘stage’ or neural circuit. Rather, they can be encoded within the neural circuit(s) performing selection, consistent with past suggestions (Cisek, 2012). Additionally, neither the norm nor its currency are intended to be identical across brain areas or universal across forms of selection. They are ‘local’ notions that ensure a common neural playing field for the comparisons being performed within a brain area at any instant.

#### Neural support

Within the literature on different forms of selection, there are several examples of neural norms that serve as the basis for comparison among options; these are discussed below.

In the context of selective attention, and specifically, selective spatial attention, the ‘priority’ of each competing stimulus is defined as the combination of its physical salience, its behavioral relevance, and potentially also the reward history associated with that stimulus (Koch and Ullman, 1987; Fecteau and Munoz, 2006; Awh et al., 2012; Figure 3B). The salience of a stimulus arises from its physical properties that ascribe inherent distinctiveness to it, such as visual contrast, speed of motion, auditory intensity (‘loudness’), etc; its behavioral relevance arises from voluntary goals associated with the stimulus such as intention to orient gaze towards it; and history-related influences arise from recent outcomes associated with the stimulus (rewarded or not) or its recent functions (distracter or not). Together, these attributes combine to represent the priority of each stimulus (in the currency of neural firing rate, for instance; Figure 3B), and result in a landscape of stimulus priorities.

Neurons in several brain areas, such as the frontal eye field (FEF) (Fecteau and Munoz, 2006), lateral intraparietal area (LIP) (Bisley and Goldberg, 2010) and in the intermediate and deep layers of the superior colliculus (SCid) across vertebrate species (Fecteau and Munoz, 2006; Mysore and Knudsen, 2011b; Knudsen, 2018), have been shown to encode stimulus priority. They exhibit a systematic relationship between firing rate and physical salience of stimuli, and respond more strongly if their preferred stimuli are behaviorally relevant. Responses of these neurons are typically insensitive to stimulus attributes that are not intrinsically salient, such as orientation or color of a visual stimulus, and frequency of an auditory stimulus. Moreover, because neurons in these areas encode sensory space topographically, stimuli (options) at different spatial locations are encoded in different parts of the topographic map. Together this leads to a spatial map of stimulus priority in which the heights (firing rates) at different locations of the map correspond to the priorities of stimuli at those locations.

In the context of value-based decision-making, options are considered to be associated with a value that depends on various attributes: magnitude of reward, time delay of reward, probability of occurrence, risk, reward expectation, ambiguity, temporal certainty, valence (appetitive or aversive), and motivation (Montague and Berns, 2002; Rangel et al., 2008; Chib et al., 2009; FitzGerald et al., 2009; Levy et al., 2010; Padoa-Schioppa, 2011; Levy and Glimcher, 2012; Yoo and Hayden, 2018). In the classical view of rational decision theory (Tversky and Kahneman, 1979; Koechlin, 2020), this value is termed the subjective expected utility (or subjective value), and is computed as the product of reward probability and magnitude. Recent work, however, indicates that this is not always the case and that reward probabilities and magnitudes can influence decisions either additively or independently (Farashahi et al., 2019; Rouault et al., 2019).

A variety of studies have demonstrated evidence for the neural encoding of different attributes in primates and rodents: of reward magnitudes (Tremblay and Schultz, 1999; Roesch and Olson, 2005; Roesch et al., 2006; Kable and Glimcher, 2007; Cohen et al., 2012; Engelhard et al., 2019), reward delay (McClure et al., 2004; Winstanley et al., 2004; Roesch and Olson, 2005; Roesch et al., 2006), risk and ambiguity (Amiez et al., 2006; Christopoulos et al., 2009; Hsu et al., 2009; Tobler et al., 2009; O'Neill and Schultz, 2010; Levy and Glimcher, 2012; Raghuraman and Padoa-Schioppa, 2014; Monosov, 2017; Chen and Stuphorn, 2018), and stimulus valence (Paton et al., 2006; Belova et al., 2007; Morrison and Salzman, 2009; Shabel and Janak, 2009; Janak and Tye, 2015). In addition, studies have also provided evidence for the (graded) neural encoding of subjective value itself (Gottfried et al., 2003; Roesch and Olson, 2005; Padoa-Schioppa and Assad, 2006; Paton et al., 2006; Kable and Glimcher, 2007; Klein et al., 2008; Schoenbaum et al., 2009; Levy et al., 2010; Plassmann et al., 2010; Sul et al., 2010; Strait et al., 2014; Sasikumar et al., 2018; Hattori et al., 2019; but see Yoo and Hayden, 2018). Notably, recent work refuting the subjective expected utility-view of value-based decision-making has revealed neural encoding of either an additive result of reward magnitude and probability, or of them, independently (Farashahi et al., 2019; Rouault et al., 2019). Typically, in all these cases, the firing rates of individual neurons (in the case of single unit electrophysiology), or the overall activation level within a patch of neural tissue (in the case of fMRI experiments), has been found to be the currency.

In the context of perceptual decisions, animals must frequently group sensory inputs into perceptual categories, with a two-category choice studied commonly in the laboratory. The categorization of a stimulus as appetitive or aversive, categorization of a complex stimulus mixture as being more similar to one or the other of its components, etc, are some of the commonly studied examples. Here, ‘degree of membership’ of a stimulus to a particular category constitutes a plausible norm.

Studies across vertebrate and invertebrate species have identified neurons in different brain areas that encode category membership. This includes encoding of the degree of belonging of a tactile stimulus to a frequency category (Romo et al., 1999; Romo et al., 2002; Brody et al., 2003; Romo et al., 2004; Hirokawa et al., 2019), of a visual stimulus to a direction-of-motion category (Freedman and Assad, 2006; Ferrera et al., 2009), an animal category (Freedman et al., 2001), or faces (Desimone et al., 1984; Freiwald and Tsao, 2010), of an auditory stimulus to a speech category (Tsunada et al., 2011) or frequency category (Znamenskiy and Zador, 2013), and of an odor mixture to one of two odor categories (Niessing and Friedrich, 2010; Olsen et al., 2010; Shen et al., 2013).

A special case within the work on perceptual decisions is the series of studies in primates involving the discrimination of the direction of coherent motion of a group of randomly moving dots (Shadlen and Newsome, 2001; Roitman and Shadlen, 2002), and in rodents involving the discrimination of the auditory stream with the greater number of clicks (Hanks and Summerfield, 2017). The conceptual formulation used to explain behavior and neural correlates in these tasks suggests another possibility for a norm in perceptual choices, namely, a ‘decision variable’. This is an abstract quantity representing at each instant, the strength of evidence in support of one perceptual choice (for instance, dots are moving to the left) versus the other (dots are moving to the right). One instantiation of this formulation involves a decision variable for each option (Usher and McClelland, 2001; Ratcliff et al., 2007) with the value of each decision variable evolving over time as evidence for that option accumulates. The rate of evolution of each decision variable (i.e., change in the magnitude of the neural response) is proportional to the strength of evidence, thereby satisfying the properties of a norm described above. Neural responses encoding evidence-dependent decision variables have been found in the monkey LIP (Shadlen and Newsome, 2001; Roitman and Shadlen, 2002), monkey FEF (Kim and Shadlen, 1999), monkey SC (Horwitz and Newsome, 1999; Ratcliff et al., 2007), monkey basal ganglia (Ding and Gold, 2012), rodent PPC and PFC (Hanks et al., 2015) and rodent striatum (Yartsev et al., 2018).

In the context of action selection, the ‘probability of execution’ of each competing motor plan constitutes a plausible norm. Studies that have examined neural activity in the presence of two competing action choices show that neurons in the primary motor cortex of primates exhibit a graded representation of each action plan depending on the probability of their selection/execution (Bastian et al., 2003; Thura et al., 2012; Thura and Cisek, 2014; Coallier et al., 2015). Additionally, neurons in the primate dorsal premotor cortex (PMd) (and the primary motor cortex) have been shown to track sensory evidence, to be modulated by the probability of execution of the motor plan (but see Dekleva et al., 2018), and to be modulated by a subjective ‘urgency’ parameter (Thura and Cisek, 2014). These data are consistent with a neurally encoded variable for each potential action that changes in a graded manner with the likelihood of that action (Cisek, 2007), thereby providing for a platform for comparing competing action plans. Indeed, when only a single action choice was presented, PMd activity did not scale with the reward associated with that choice, consistent with the idea that PMd activity encodes for probability of selection (Pastor-Bernier and Cisek, 2011).

Thus, there is support from different forms of selection for the representation of competing options in a graded manner that reflects their norm. In many cases, there is additional support for the norm being unidimensional, and for it being encoded in the common currency of neuronal firing rates. This, then, sets the stage for the first step in the computational framework for competitive selection.

### Comparison

#### Conceptual set-up

The first crucial computational feature of the WTA operation that must be implemented by neural circuits is comparison among competing options, that is, the ‘>’ operation (Equation 1a).

#### Neural circuit requirements

Comparison among options can be achieved in neural circuits through inhibition. This can be done by having each option evoke inhibition that is proportional to its norm, and that modulates the representations of all options. Notably, the anatomical scale of the inhibition must be ‘global’, able to reach all the neurons that encode potential competing options. This allows each option to be compared against the others, effectively producing representations that reflect the *relative* norms of the competing options. From an implementational perspective, one straightforward way to achieve this is via global feedforward inhibition (Figure 4). Such feedforward inhibition among the representations of the options normalizes inputs based on total drive, ensuring that responses stay within the neural dynamic range (Olsen et al., 2010). (We note, however, that this is not the only way to implement comparison, we will discuss an alternate, feedback, implementation in a subsequent section titled ‘Generality: Comparison …’).

#### Neural support

Examples of inhibition sub-serving comparison and selection have been reported in different brain areas and for several forms of selection. Reports involve either the indirect inference of neural inhibition from response patterns - typically by comparison of responses when one option is presented with those when multiple options are, or a direct demonstration of it through causal experiments involving the silencing of appropriate (inhibitory) neurons.

In the context of selective (spatial) attention, studies have demonstrated a suppression of neural responses to a stimulus inside the RF when a distant competitor is also simultaneously presented, leading to the plausible inference that neural inhibition plays a key role (Rizzolatti et al., 1974; Frost et al., 1981; Falkner et al., 2010; Mysore et al., 2010). This inference is strengthened by studies that have tested separately conditions that produce an enhancement versus those that produce a suppression (Winkowski and Knudsen, 2008), as well as studies in behaving animals in which it was possible to infer response suppression unambiguously (Gazzaley et al., 2005).

In addition to these indirect indications of competitive inhibition, studies in birds have silenced candidate inhibitory neurons in the midbrain tegmentum and directly demonstrated the presence of competitive inhibition in selection underlying spatial attention (Marín et al., 2007; Mysore and Knudsen, 2013). Taken together, these studies provide support that the brain uses competitive inhibition for comparison and selection in the context of (spatial) selective attention.

In the context of value-based decision-making, competitive inhibition has been proposed to explain neural responses in the monkey OFC during a choice between two goods (Ballesta and Padoa-Schioppa, 2019). Comparisons among neurally encoded choice values have also been proposed to occur in the ventral striatum, mPFC and posterior cingulate cortex in humans (Kable and Glimcher, 2007), and in the ACC in monkeys (Hayden et al., 2011; Rushworth et al., 2012). However, inhibitory neurons that might sub-serve competition in value-based decision-making have not yet been identified.

In the context of perceptual categorization, in tasks in which animals have to make a forced choice based on a noisy stimulus, neurons in the primate LIP (Shadlen and Newsome, 2001; Roitman and Shadlen, 2002), rodent parietal cortex (Hanks et al., 2015), primate PFC (Kim and Shadlen, 1999), rodent PFC (Erlich et al., 2011; Hanks et al., 2015) and rodent striatum (Yartsev et al., 2018) show ramping activity that is enhanced if evidence in the stimulus favors the neuron’s preferred choice but is suppressed if the evidence favors the alternate choice. These data provide support for the hypothesis that activities of neurons accumulating evidence for different choices are compared, potentially through competitive inhibition. In *Drosophila*, feedforward (lateral) inhibition has been shown to play a key role in odor discrimination (Olsen et al., 2010).

Similar results have been found in delayed match-to-sample versions of perceptual decision-making tasks, in which animals were required to report whether a test stimulus had a higher or lower value of a particular feature, when compared to that of an earlier reference stimulus. A common finding is the presence of neural sub-populations with enhanced firing rates when the reference stimulus is larger in the feature being tested than the test (or in the same category as the test), as well as other neurons with a complimentary pattern of firing, suggesting a competitive comparison between the two stimuli: in S2 (Romo et al., 2002), DLPFC (Romo et al., 1999), ventral premotor areas (Brody et al., 2003; Romo et al., 2004), PFC (Freedman et al., 2001; Ferrera et al., 2009), LIP (Freedman and Assad, 2006) and inferior temporal cortex (ITC) (Freedman et al., 2003). Although, direct evidence of where and how the comparison and inhibition happens is not yet known, models have hypothesized the presence of inhibitory interactions between the option representations (Gold and Shadlen, 2007; Tsunada and Cohen, 2014).

In the context of action selection, consistent with early proposals (Jeffress, 1951; Bullock, 2004), there is evidence that multiple competing motor plans are readied in parallel preceding the selection of just one (Cisek and Kalaska, 2005; Cui and Andersen, 2007; Seeds et al., 2014; Dekleva et al., 2016; Hampel et al., 2017; but see Dekleva et al., 2018). In these cases, over the course of action selection, the activity representing the chosen plan is enhanced while the other competing plans are suppressed. This pattern of responses has been explained by the proposal that competing actions interact with each other through inhibition, via mechanisms that are yet to be discovered (Cisek and Kalaska, 2005; Thura and Cisek, 2014). In the context of complex (hierarchical) action programs, several studies have strongly suggested inhibition between competing behavioral modules. In the marine mollusk, rhythmic feeding behavior and a withdrawal response compete, with bilaterally symmetric interneurons in the buccal cavity being implicated in this competition (Kovac and Davis, 1977; Kovac and Davis, 1980). In *Drosophila*, a suppression hierarchy of motor programs for smaller stereotyped cleaning movements has been identified as underlying grooming behavior (Hampel et al., 2017), with potentially (asymmetric) inhibition between competing modules (Edwards, 1991; Seeds et al., 2014; Hampel et al., 2017).

In parallel, direct evidence for the involvement of competitive inhibition among multiple action plans has been reported in studies across species: zebrafish (Koyama et al., 2016), *Drosophila* larvae (Jovanic et al., 2016), and mice (Fadok et al., 2017; Hong et al., 2014).

In summary, based on studies of the neural correlates of selection, there is evidence for (norm-dependent) inhibition involved in the comparison among options. Direct evidence for the presence of such inhibition has been demonstrated in a subset of the cases. Going forward, it will be important to experimentally identify the source of competitive inhibition, and to demonstrate its computational contribution, in various brain areas underlying the different kinds of selection.

#### What can the circuit thus far NOT do?

The circuit depicted in Figure 4 can account well for selection of the best option when the two options presented are significantly different from one another. However, when the options are close to one another in norm, it is not effective at signaling the winner reliably, especially in the presence of neural response variability (Carandini and Churchland, 2013; Mahajan and Mysore, 2020). We turn to this computational feature next.

### Categorical selection boundary

#### Conceptual set-up

The next ‘hidden’ computational feature of the idealized WTA operation is the categorical identification of the option with the highest norm. For instance, in a two-input example in which the first one has a higher value, the idealized WTA operation correctly selects it as the winner both when the inputs are [10, 2], that is when *x_1_* has a much higher value than *x_2_*, and when the inputs are [10, 8], that is when there is only a small difference between them. In other words, the idealized WTA operation implements a step-like category boundary.

In practical terms, the ability of neural circuits to approximate a step-like function promotes the selective enhancement of response differences between similar options straddling the selection boundary, thereby improving the reliability of the signaling the winning option. This improvement in reliability can be appreciated particularly in the context of neural 'noise' (or response variability) that is ubiquitous in biological circuits. If, for instance, the norms of two competing options *x_1_*, and *x_2_* are represented by noisy firing rates [10 ± 0.8, 8 ± 0.6] spikes/sec (mean ± sd), the difference between the firing rates on any individual trial can be very small. On the one hand, biological circuits rarely produce a perfectly step-like nonlinearity. On the other hand, a circuit mechanism that can selectively enhance differences across the selection boundary to produce explicitly categorical outputs from continuous inputs (Gollisch and Meister, 2010) can improve signaling reliability substantially. (We note that our discussion here relates specifically to neural responses, and does not impose a requirement that behavioral response profiles be step-like.)

#### Neural circuit requirements

At first glance, amplifying the responses to all options multiplicatively (i.e., with a gain factor) may seem like a potential mechanism to enhance differences between their norms. However, in the context of noisy (neural) responses, the difference between the norms of two options is more reliably quantified by discriminability (d’=difference in response means/√(average response variance)), rather than by a simple disparity between the means. Because uniform amplification scales up both the mean as well as the standard deviation equally, it does not help improve the discriminability. What is needed instead, is differential amplification, such that responses to options that just straddle the selection boundary are driven apart. In simple terms, the more categorical the representation across the selection boundary, the more robust-to-noise the selection will be (Figure 5A).

Recently, it was demonstrated that a donut-like inhibitory motif, in which each option suppresses the representation of all options except its own (Figure 5B), is highly effective at generating categorical selection boundaries (Mahajan and Mysore, 2020). It is also substantially more effective than other commonly invoked motifs in decision-making models, namely - recurrent amplification, feedback inhibition and divisive normalization. (This motif as well, can be implemented either via feedforward inhibition or feedback inhibition, as demonstrated in Mahajan and Mysore, 2020; also see section titled ‘Generality: Comparison…’).

A second mechanism that has been proposed, specifically in the context of decision-making in circular feature spaces (for instance, the space of motion directions, or orientations), is structured synaptic inhibition (Xue and Liu, 2014). In this scheme, inhibitory neurons encoding for a particular feature value (say, motion direction) deliver strong feedforward inhibition to the excitatory neurons that encode either the same motion direction or the opposite motion direction, but weak inhibition, to excitatory neurons encoding for all other orientation values. In addition, these inhibitory neurons suppress one another (and themselves) with a uniform strength (Figure 5C). It is unclear whether this mechanism directly generalizes to non-circular feature spaces.

#### Neural support

In the literature, a host of studies have found categorical neural representations in the context of different forms of selection. However, only one study thus far has investigated experimentally how this is achieved in a neural circuit (Mahajan and Mysore, 2020). We discuss these points below.

In the context of selection for spatial attention, explicitly categorical responses in the sensorimotor pathway have been reported, to date, only in the optic tectum of the barn owl (OT, or superior colliculus in mammals) (Mysore et al., 2011; Mysore and Knudsen, 2011b; Mysore and Knudsen, 2014). In the other classic hubs along the oculomotor pathway, including the pulvinar (Saalmann et al., 2012), LIP (Gottlieb et al., 1998), FEF (Sato et al., 2001; Thompson and Bichot, 2005), and rodent visual thalamus (Wimmer et al., 2015), responses to the target have been shown to be higher than those to distracter(s), but have not been shown to be explicitly categorical -- likely a consequence of the specific protocols used to probe selection and of the lack of appropriate parametrization of the stimuli (Box 2). Interestingly, in one primate study that did use a stimulus protocol close to the one used in the owl study, response profiles of FEF neurons in a stimulus-speed categorization task were found to be nearly linear as a function of the stimulus speed (Ferrera et al., 2009). These markedly non-categorical response profiles were, however, shown to implicitly (Gollisch and Meister, 2010) contain information that could be used by downstream neurons to create categorical responses. Thus, whether other hubs (beyond the avian OT) in the vertebrate visuospatial attention pathway encode explicitly categorical responses in the context of spatial selection and attention is to be determined.

Box 2. Measurement of neural correlates of WTA competitive selection.

A convincing demonstration of the neural correlates of the WTA operation requires that neural responses exhibit the following properties:

1. That the neural responses to the selected option are substantially and categorically different from those to the other competing options, or in other words, that a clear winner is explicitly evident in the neural responses, rather than having to be inferred by applying additional nonlinear transformations to the responses (Gollisch and Meister, 2010; Figure 5A: Leftmost panel represents implicit signaling of the winner; rightmost panel represents explicit signaling of the winner).
2. That the selected option ‘takes-all’, or in other words, that the neural responses to the non-selected or ‘losing’ options are driven either to zero (‘hard’ WTA), or to a non-zero level that is below the threshold for triggering an output action or percept (‘soft’ WTA).
3. That (1 - 2) hold not just when the competing options differ from each other substantially in terms of their norms but also when they have similar norms. In other words, these properties must hold under systematic, parameterized variation of the norms of the competing options.
4. In addition, that (1 - 3) hold, in general, for all pairs of competing options, and also when the number of competing options is varied.

From a mechanistic perspective, a recent study (Mahajan and Mysore, 2020) built on previous anatomical work in reptiles and birds (Sereno and Ulinski, 1987; Wang et al., 2004; Gruberg et al., 2006), and used selective inactivation experiments in barn owls to investigate the mechanism of inhibition underlying categorical selection in the optic tectum (OT). The study demonstrated not only that the pattern of net competitive inhibition received by the optic tectum is functionally donut-like, but also that the donut-like pattern is required for categorical signaling by the OT (Mahajan and Mysore, 2020).

In the context of value-based decision-making, studies have shown that animals are able to select categorically between options based on a subjective boundary (see also Yoo and Hayden, 2018). In some cases, for instance, in a task involving selection between two different juice rewards, neurons in the primate OFC have been shown to encode categorical correlates of choice (the ‘taste’ coding neurons; (Padoa-Schioppa and Assad, 2006; Onken et al., 2019). In a recent study involving a sequential offer task (Ballesta and Padoa-Schioppa, 2019), a modeling approach suggests the need for inhibition between the options with a pattern that is reminiscent of the donut-like motif (Figure 5B); the actual implementation of this inhibition within cortical circuits is yet to be tested. In other cases, for instance, in the vmPFC, responses during value-based decision-making tasks have, thus far, not been shown to exhibit categorical profiles (Kable and Glimcher, 2007; Hunt et al., 2012; Hunt et al., 2013; Strait et al., 2014). However, because these studies did not vary parametrically the relative values of reward options, a requirement for assessing whether response profiles are categorical, it is possible that the appropriate experimental design will reveal categorical signatures of selection (Box 2). Thus far, no explicit circuit level solution for categorical representations underlying value-based decision-making has been identified experimentally.

In the context of perceptual decision-making, categorical neural responses have been reported in numerous studies across brain areas. These include the encoding of complex sensory stimuli (mixtures) (Roitman and Shadlen, 2002; Heekeren et al., 2008; Niessing and Friedrich, 2010; Olsen et al., 2010; Freedman and Assad, 2016), responses to auditory note identities (Prather et al., 2008), and responses in delayed match-to-category and match-to-sample tasks in which an animal has to make a categorical decision regarding either whether two temporally separated stimuli belong to the same category or whether the second stimulus is greater or less than the first one, respectively. For the latter group of tasks, neurons in primate LIP (Freedman and Assad, 2006), premotor cortex (Hernández et al., 2002; Romo et al., 2004), S2 (Romo et al., 2002) and PFC Freedman et al., 2001 have been shown to exhibit categorical responses. Just as with value-based decision-making, many models of perceptual categorization include a general mutual inhibition architecture (Roe et al., 2001; Usher and McClelland, 2001; Machens et al., 2005; Bogacz et al., 2007), which intrinsically admits the donut-like inhibitory motif (Mahajan and Mysore, 2020; Figure 2C. see section titled ‘Generality: Comparison…’).

There is also some support for the alternative mechanism of structured synaptic inhibition (Xue and Liu, 2014), which was proposed to account for behavioral results and LIP activity during motion-direction discrimination (Churchland et al., 2008). Inhibition between neurons encoding for similar feature values (motion direction or orientation) as well between neurons encoding opposite feature values has been inferred in early sensory cortices (Ringach et al., 1997; Neri and Levi, 2009). Whether similar mechanisms are at play in the LIP and whether they underlie robust-to-noise selection, in general, are not yet known.

In the context of action selection, models proposed in several studies have included mutual inhibition – in primates (Cisek and Kalaska, 2005; Cisek, 2006; Pastor-Bernier and Cisek, 2011; Thura and Cisek, 2014), as well as in invertebrates (Kovac and Davis, 1977; Kovac and Davis, 1980; Edwards, 1991; Kristan, 2008; Seeds et al., 2014; Hampel et al., 2017), but no neural circuit details have been offered, leaving open the question of where and how categorical responses are implemented. In other studies in which neural circuits involved in action selection tasks have been identified, for instance, in selection between orienting responses (Koyama et al., 2016), and between more complex behavior programs (Hong et al., 2014; Jovanic et al., 2016; Fadok et al., 2017), the identified circuit architectures are consistent with the donut-like motif. However, experimental evidence for the computational contribution of the donut-like circuit motif in these tasks is yet to be reported.

In summary, there is strong support for categorical neural representations underlying different forms of selection. Additionally, the operation of the proposed donut-like inhibitory circuit mechanism for robust-to-noise selection has been experimentally demonstrated in the avian midbrain in the context of selection for spatial attention (Mahajan and Mysore, 2020). For the other forms of selection (and brain areas), although many computational proposals in the literature are consistent with this circuit motif (and more so than with the structured inhibition motif), an experimental demonstration of the circuit mechanism for categorical selection in those cases remains an open question.

An intriguing issue in this context is that whereas categorical neural representations are pervasive, behavioral responses are frequently less categorical: psychometric performance curves (for instance, % correct curves), typically vary gradually with the independent variable (but see You and Mysore, 2020. A plausible explanation is that behavior is the consequence of the aggregated response of a large population of neurons with the animal as a whole frequently performing worse than individual neurons (Newsome et al., 1989). This, however, does not take away from the fundamental issue of how categorical neural representations are produced. It also raises the question of what might be the effect on neural and behavioral responses of disrupting the underlying circuit mechanism? A testable prediction is that such a disruption would cause psychometric curves to become even less categorical than normal, worsening behavioral performance around the selection boundary.

#### What can the circuit thus far NOT do?

The circuit depicted in Figure 5 implements a fixed (categorical) selection boundary, with the value being determined by the biophysical properties of the neurons (input/output functions, synapses etc; Mysore and Knudsen, 2012). Therefore, if the options encountered by animals require the selection boundary to shift to different values dynamically, this circuit would be incapable of doing so (Mysore and Knudsen, 2012). This computational feature is considered next.

### Dynamic flexibility of the selection boundary

#### Conceptual set-up

The third ‘hidden’ computational feature of the WTA operation is the identification of the option with the largest norm (i.e., the ‘winner’), *independently* of the absolute values of the norms of the options. For instance, in a selection problem involving two inputs x_1_, and x_2_, WTA correctly selects the winner x* as the first option (x* = x_1_) both when the inputs are, respectively, [9, 5], and when they are [20, 13]. A fixed boundary would not work in both cases, but instead, a category boundary of 9 is needed in the first case, and of 20, in the second.

In practical terms, this is a problem that animals, and in turn, their neural circuits, can encounter on a regular basis. For instance, the absolute priorities of a pair of competing potential targets of attention can be quite different at two different instants, or the amounts of juice reward associated with two options can differ between trials. To select the winning option consistently, the selection boundary cannot be fixed at a pre-determined value, but rather, needs to be flexible, adjusting to the set of options available at any instant (Figure 6A).

The dynamic nature of such flexibility, which requires the generation of flexible selection boundaries on the fly, places the constraint that the underlying circuit implementation cannot rely upon plasticity mechanisms. This is in contrast to the slower form of learned flexibility that has been studied in the decision-making literature (Bichot et al., 1996; Miller and Cohen, 2001; Mante et al., 2013; Dajani and Uddin, 2015; Nilsson et al., 2015). There, the response expected to the same stimulus can be qualitatively different in different contexts, based on response 'rules' that are learned by experience and then invoked flexibly based on contextual cues (Freedman and Assad, 2006; Jaramillo et al., 2014; Xiong et al., 2015). Such rule-based flexibility is thought to involve various mechanisms including synaptic plasticity in the appropriate neural pathways (Xiong et al., 2015) and astrocyte function in the cortex (Brockett et al., 2018). By contrast, the ‘flexible category boundary’ that we refer to here, requires flexibility to be built-into the underlying circuitry.

#### Neural circuit requirements

Recent computational modeling (in the context of selection for spatial attention) has predicted that the circuit motif necessary for achieving flexible selection boundaries is feedback inhibition between the representations of the competing options (Mysore and Knudsen, 2012). It stands opposed to feedforward inhibition, the magnitude of which is not modulated by its effect on downstream targets, and which is has been shown to be insufficient for flexibility (Mysore and Knudsen, 2012). Modeling demonstrates that the reciprocal inhibition of inhibition motif (Figure 6B) is the most efficient implementation of feedback among the many implementations that can all work (Mysore and Knudsen, 2012; Figure 6DE). (Incidentally, the terms ‘mutual inhibition among competing options’ or ‘lateral inhibition’ used in previous studies do not always disambiguate between the feedforward vs. feedback scenarios described here. For this reason, we prefer the use of the terminology of feedback as opposed to feedforward inhibition.)

#### Neural support

Reciprocal inhibition of inhibition has been reported in several brain areas (Pangratz-Fuehrer and Hestrin, 2011; Picardo et al., 2011), including within networks that are involved in different forms of flexible selection (Deleuze and Huguenard, 2006; Brown et al., 2014; Goddard et al., 2014; Jovanic et al., 2016; Fadok et al., 2017; Koyama and Pujala, 2018).

In the context of spatial attention, the reciprocal inhibition of inhibition motif has been found within the avian Imc (Wang et al., 2004), a GABAergic midbrain tegmental nucleus that is critical for stimulus selection across space (Marín et al., 2007; Mysore and Knudsen, 2013): Imc neurons that encode for distinct locations in space functionally inhibit one another with long range connectivity (Goddard et al., 2014). In mammals, the thalamic reticular nucleus is known to play a key role in sensory selection (Nakajima et al., 2019), and the reciprocal inhibition of inhibition motif has been reported within it (Deleuze and Huguenard, 2006). However, the contribution of these feedback inhibitory motifs to flexible selection for attention is yet to be demonstrated experimentally.

In the context of value-based decision-making, studies have recently started to investigate the underlying neural circuit mechanisms (Ballesta and Padoa-Schioppa, 2019), and the neural basis of dynamic flexibility has not yet been investigated explicitly.

In the context of perceptual decision-making, several studies have measured neural responses that support flexibly categorical representations. For instance, in delayed match-to-sample tasks involving tactile stimuli, responses in the premotor cortex (Hernández et al., 2002; Romo et al., 2004), S2 (Romo et al., 2002) and PFC (Brody et al., 2003) are consistent with a flexible section boundary that facilitates the identification of whether the test stimulus was of greater frequency than the reference. Similarly, in a delayed match-to-sample task involving visual stimuli, responses in the monkey frontal eye field (FEF) have been shown to contain information to allow downstream neurons to create flexible and explicit categorical output (Ferrera et al., 2009). In addition, computational models that have been proposed to account for the observed responses underlying flexible selection behavior have included mutual (feedback) inhibitory interactions between the competing options (Machens et al., 2005; Wong and Wang, 2006). However, where in the brain, and how, flexibility is implemented in the context of these tasks is unclear. In the context of odor representations, feedback inhibition is known to exist between glomeruli in the olfactory bulb (Li et al., 2019), which may allow for flexibility in odor discrimination.

In the context of action selection, the reciprocal inhibition of inhibition motif has been reported in several studies – in the hindbrain (Mauthner cell circuit) of larval zebrafish for left versus right escape behavior (Koyama et al., 2016), in the projection neurons of *Drosophila larvae* for selection between startle and exploratory behavior (Jovanic et al., 2016), and in the central amygdala of mice for selection between passive freezing and conditioned flight following fear conditioning (Fadok et al., 2017). Whereas silencing inhibitory neurons in all these studies has directly demonstrated a role for them in selection, the specific computational role for feedback inhibition has not been demonstrated directly.

In summary, despite the proposed essential role of feedback inhibition for flexible selection, and the presence of this motif in several brain areas/selection circuits, its necessity for flexibility of selection boundaries is yet to be tested experimentally.

#### What can the circuit thus far NOT do?

Flexible, categorical selection within a single pair of options is insufficient for adaptive behavior. A circuit for selection must be able to select between any given pair of options. The circuit in Figure 6 will need to be elaborated to achieve this computational feature, and this is considered next.

### Ability to select among many (all) viable pairs of options

#### Conceptual set-up

The fourth ‘hidden’ computational feature of the idealized WTA operation is the identification of the winner no matter which specific inputs {*x_k_*} are active at any instant (and which of those happens to be the largest).

In practical terms, selection and decision-making are typically versatile, operating over a wide array of potential options. For instance, one can select one’s preferred fruit between oranges and apples, between blueberries and pomegranates, and so on. Similarly, selection for spatial attention operates for stimuli occurring across a large collection of possible locations. Competitive selection must, therefore, function for every viable pair (group) of options, despite different options activating distinct groups of neurons (or neural 'channels') (Figure 7A; top vs. bottom panels). Without this ability, we would be able to select among only some fruit pairs but not others, and attend to a target only when competing stimuli are presented at some location pairs but not at others.

#### Neural circuit requirements and support

To implement this computational feature, the underlying neural circuitry must be designed to handle comparisons for different (all possible) pairs of evoked neural representations.

In the context of selection for spatial attention, two strategies that neural circuits could employ to this end were presented recently (Mahajan and Mysore, 2018). The first is a copy-and-paste strategy (Figure 7A), in which the circuit module required to compare one pair of options and select between them is simply reproduced as many times as needed to serve all possible pairs of potential channels. The second strategy, which was discovered experimentally to be in operation in the barn owl midbrain, is combinatorially optimized coding (Figure 7B; Box 3; Mahajan and Mysore, 2018). Briefly, the authors discovered that inhibitory neurons that underlie spatial selection in the midbrain attention network, namely Imc neurons, are sparse in number, encode sensory space densely (with multi-lobed RFs), and are activated combinatorially in order to deliver competitive inhibition at all pairs of stimulus locations (Box 3). Theory and modeling demonstrated that this strategy, termed COSMI (**c**ombinatorially **o**ptimized coding by **s**parse, **m**ultilobe **i**nhibitory neurons), is highly efficient compared to the brute-force copy-and-paste strategy when high-firing neurons (in this case, parvalbumin-positive neurons) are involved – it minimizes the combined metabolic and wiring costs involved in operating the selection circuit.

Box 3. Ability to solve selection at all pairs of stimulus locations: the COSMI strategy (Combinatorially Optimized feature coding by Sparse, Multilobed Inhibitory neurons).

Consider that there are L possible spatial locations at which stimuli could occur in the animal’s representation of the environment. Then, the neural circuit underlying stimulus selection across space must be capable of comparing and selecting for each of the L(L-1) possible pairs of locations. A straightforward solution to this problem is a modular copy-and-paste strategy (Mahajan and Mysore, 2018; Figure 7A), in which the circuit module required to compare one pair of options and select between them is reproduced as many times as needed to serve all possible pairs of potential locations.

However, since the number of possible pairs of locations varies as L^2^, this copy-and-paste strategy places heavy demands on the costs of building and operating the circuitry, namely on wiring and metabolic costs. Therefore, it is unclear *apriori* whether the modular copy-and-paste strategy is preferred by the brain or whether an alternate strategy involving optimized connectivity and specialized encoding principles are biologically preferable.

This was recently addressed in the context of selection for spatial attention in the barn owl midbrain (Mahajan and Mysore, 2018; Figure 7B). The authors investigated the functional logic of a group of inhibitory midbrain tegmental neurons, the Imc, which are known to play a critical role in selection across space (Marín et al., 2007; Mysore and Knudsen, 2013). The study discovered that Imc neurons are sparse in number, and employ a combinatorial strategy in order to deliver competitive inhibition at all pairs of stimulus locations (Figure 7B; Mahajan and Mysore, 2018). To support this strategy, Imc neurons encode spatial locations with unusual receptive fields (RFs) with multiple discrete hotspots or lobes, thereby producing a dense coding of space (Figure 7C). Notably, the RF lobes of *individual* neurons are carefully optimized *across* the Imc population. This strategy was shown to minimize the sum of the metabolic costs of spikes generated by the high-firing Imc neurons, and the wiring costs of building the donut-like circuit (Mahajan and Mysore, 2018). Thus, inhibitory neurons in the owl midbrain implement selection at all pairs of spatial locations in the space map of the optic tectum, and do so optimally by using a small number of high-firing inhibitory neurons that encode space in a multilobed manner such that they are activated in a combinatorial fashion by competing stimuli (Mahajan and Mysore, 2018).

In the context of perceptual categorization, the use of different odor mixtures has demonstrated that selection is indeed versatile, capable of occurring among many distinct pairs of options (Niessing and Friedrich, 2010). In value-based decision-making, a recent study Xie and Padoa-Schioppa, 2016 found that when monkeys were presented with two different sets of value-based choices (A vs B, or C vs D), the neurons in the OFC encoding the decision variable remained stable across contexts. This suggested a remapping at the earlier evaluation stage (Xie and Padoa-Schioppa, 2016), although potential mechanisms such remapping are as yet unclear. It is plausible that the remapping, which involves routing activity related to the preferred option (among competing ones) to an assigned neural population, itself occurs via a competitive process.

Outside of the owl study (Mahajan and Mysore, 2018), the circuit mechanisms underlying such ‘versatile’ selection have not been identified. Nonetheless, the seemingly specialized strategy employed by owl Imc neurons has broad conceptual appeal owing to the relatively straightforward constraints that yield it: the involvement of inhibitory neurons with feedback and donut-like connectivity, whose function is additionally governed by the plausible principle of minimization of net neural costs. Such a combinatorial scheme is consistent with the patterns of suppression reported in olfactory bulb glomeruli in mice (Economo et al., 2016).

Thus, versatility of selection across many distinct option-pairs is a useful computational feature. Whether the circuit strategy of combinatorially optimized inhibition is also found in other brain areas/species, or more generally, what the strategy might be, for other forms of selection is yet to be determined experimentally.

#### What else ought a selection circuit be able to do?

Flexible, categorical signaling among all viable pairs of options leads naturally to the next feature; selection among more than two options at any instant. This computational feature is considered next.

### Ability to select among multiple (>2) options

#### Conceptual set-up

The computations we have discussed, thus far, have all dealt nominally with two competing options at a time. However, the natural world is rich with potential options, and animals frequently select among more than just two competing alternatives. It is, therefore, useful for neural circuits to be able to handle selection amidst such complexity in order to facilitate adaptive behavior. Indeed, this ability is the fifth hidden computational feature of the idealized WTA equation: the winner x* among multiple competing options *{x_i_}* is correctly identified even when many *x_i_* are present (i.e., *i* > 2).

An understanding of the neural circuit requirements underlying selection among multiple options is still in its infancy (Churchland and Ditterich, 2012). To motivate better the discussion about potential circuit mechanisms, we first summarize key results from relevant neurophysiological experiments and associated models, and then consider circuit implications.

#### Neural and behavioral data

In the context of selection for spatial attention, several primate studies have used more than one distracter in conjunction with the attentional target. They have shown that responses of neurons in the FEF (Lee and Keller, 2008; Cohen et al., 2009), LIP (Balan et al., 2008), and midbrain superior colliculus (SC) (Basso and Wurtz, 1997; Basso and Wurtz, 1998), to the target stimulus decrease with increasing number of distracters. Recently, work in the barn owl has begun exploring the effect of the number as well as relative priorities of distant competitors on different aspects of competitive responses in the optic tectum (Rajagopalan et al., 2018). Results from increasing the number of competitors are consistent with primate results. Broadly, a role for inhibition that is dependent on the priorities of the stimuli (rather than just their number) has been suggested as a potential mechanism (Basso and Wurtz, 1997; Churchland and Ditterich, 2012).

In the context of perceptual decision-making, responses of neurons in the monkey parietal cortex have been investigated during a task in which they had to report the direction of a noisy motion stimulus when presented with either two, or four response options (Churchland et al., 2008; Churchland et al., 2011). Similar to the studies of spatial attention, this study reported a reduction in neural activity in the LIP (and an increase in response variability) when the number of available options increased. Nonetheless, at the time of the decision, neural firing rates were similar, suggesting that the final threshold value for choice may be constant, with the underlying network requiring more evidence and longer time to reach that threshold. In addition, behaviorally, preference reversal and reference effects have been reported in the context of selection among multiple alternatives (Mellers and Biagini, 1994; Roe et al., 2001; Usher and McClelland, 2004).

In the context of value-based decision-making, neural responses in the primate LIP to multiple options also show similar reduction in activity with increasing number of options (Louie et al., 2011).

Finally, in the context of action selection, neural correlates in the presence of >= 2 movement options have been examined (Bastian et al., 2003). As in other forms of selection, firing rates in PMd have been shown to decrease with increase in target uncertainty (i.e., increase in number of options) (Dekleva et al., 2016), and in M1, a measure corresponding roughly to response variability has been shown to increase systematically (Bastian et al., 2003).

#### Models and neural circuit requirements

Several variants of A2T and WTA models have been proposed to explain perceptual decision-making (Roe et al., 2001; Usher and McClelland, 2004; Bogacz et al., 2007; Furman and Wang, 2008; Churchland and Ditterich, 2012; Deco et al., 2013; Xue and Liu, 2014; Tajima et al., 2019), value-based decision-making (Glimcher, 2014) and action selection (Cisek, 2007; Cisek and Kalaska, 2010) amongst multiple options.

On the one hand, they account for key neural and behavioral effects in animals – reduction in firing rates, changes in RTs and reduced accuracy, and in addition, account for key effects on behavioral choice observed in humans, namely, reference, context-dependent preference reversal, and set-size effects (Tversky and Kahneman, 1991; Tversky and Simonson, 1993; Wolfe, 1994; Roe et al., 2001; Usher and McClelland, 2004).

On the other hand, the proposed models have a range of differing circuit design details - involving feedback versus feedforward inhibition, involving linear versus nonlinear mechanisms for combining signals across alternatives, involving pooled versus option-specific (lateral) inhibition that is value-dependent, structured versus homogeneous inhibitory connectivity (across feature space), etc (Roe et al., 2001; Usher and McClelland, 2004; Bogacz et al., 2007; Furman and Wang, 2008; Deco et al., 2009; Ditterich, 2010; Bollimunta and Ditterich, 2012; Churchland and Ditterich, 2012; Xue and Liu, 2014; Murakami and Mainen, 2015; Tajima et al., 2019). Because of this diversity, it is unclear which implementational aspects of these models are essential for handling computations underlying selection among multiple stimuli, and which ones are not. Notably, because the models are direct extensions of their two-option counterparts, it would appear that there are no systematic additional constraints that come into play specifically when multiple options, rather than just two, are to be dealt with.

Recent experimental and modeling work in the barn owl suggest otherwise. Specifically, examination of the flexibility of selection boundaries suggests that additional constraints emerge when selection is generalized to beyond just two options, and that a direct extension of two-option models may be insufficient (Rajagopalan et al., 2018). Preliminary results indicate that the core issue may relate to the manner in which inhibition from all other options is combined to affect the representation of each option, and that this may need to be nonlinear (Figure 8A; yellow oval). Notably, this requirement of nonlinearity is consistent with one series of models of multialternative decision-making (Bogacz and Gurney, 2007; Bogacz et al., 2007; Zhang and Bogacz, 2010).

In summary, key issues underlying selection among multiple options may yet need to be resolved experimentally. Some open questions are: ‘how does inhibition scale with the number of options?’, ‘how is inhibition from multiple options combined?’, ‘how are floor effects avoided (to ensure that neural responses to the winner stay robust and are not washed out despite inhibition from an increasing number of options)?’, and ‘how do the computational features of flexibility and categorical representations, which have been discussed thus far in the context of two options, continue to be accomplished with >2 options?’.

### Unitary output generation

#### Conceptual set-up

Following the flexible and categorical identification of the winner within any set of competing options, the final (and an essential) computational feature of selection involves ensuring that only the winning option, but not any of the others, is capable of triggering an action or percept. This is implicit in the idealized WTA equation (Equation 1a), which sets the winning option to a high value but all the non-winning options to zero (by definition).

From the point of view of neural circuits, this computational feature translates to a slightly less stringent scenario. Neural circuits do need to ensure that the responses to the winning option are above the functional threshold of output-generating neurons (Lo and Wang, 2006; Furman and Wang, 2008; Seger and Peterson, 2013). However, responses to the other options do not necessarily need to be driven to zero, they just need to be weaker than this functional response threshold. (We note, here, that unitary choice formation is essential only at the very final stage in the selection process, just prior to output production, but is not required to occur at any of the previous stages or sites of computation in the brain.)

#### Neural circuit requirement

We propose that this computation can be implemented in neural circuits by incorporating two additional features – by making the gain of the competitive inhibition high, and by coupling that with recurrent amplification of each option’s representation.

The logic is as follows. Competitive inhibition evoked by each option is required, based on the discussion thus far, to be proportional to that option’s norm, and to produce a donut-like output pattern. If the gain of this inhibition were to be set to a high value, this would have the desired consequence of driving the responses of low-norm options to very low values. However, this raises the potential concern that the high inhibitory gain could cause the losing options to drive the winner’s responses to values that may be too low to be effective for driving output. Conceptually, this concern can be offset with an amplification mechanism that preferentially scales the representation of just the winner. This can be implemented by a bank of recurrent amplifiers, one for each option, positioned such that each amplifier also receives the high-gain competitive inhibition (Figure 8BC). This second feature will facilitate preferential amplification of just the highest-norm option, because the remaining amplifiers in the bank, corresponding to all other options, are powerfully inhibited by the high-gain competitive inhibition.

The recurrent excitation required by this implementation is a classic mechanism that has been proposed previously for the accumulation of evidence over time (Furman and Wang, 2008; Wang, 2008; Wang, 2012). As a result, By virtue of being framed in the WTA perspective, the above computational feature, and all the ones so far, have been discussed nominally as operating on steady-state firing rate responses. On the other hand, responses in neural circuits evolve over time, and animals make decisions under temporal urgency (Reddi et al., 2003; Churchland et al., 2008; Thura et al., 2012). It is, therefore, important to note that the circuit proposed above is well capable of producing temporally evolving responses seen in the brain, and in A2T models: The recurrent excitation required in this implementation is, in fact, a classic mechanism that has been proposed for the accumulation of evidence over time (Furman and Wang, 2008; Wang, 2008; Wang, 2012). (In addition, the non-selective motor urgency signal, which is thought to multiplicatively modulate the sensory input (Thura et al., 2012), is plausibly accounted for as one of the (many) internal influences that go into building the representations of the options; Figure 1.) Therefore, with the proposed circuit mechanism for unitary choices, responses to the competing options are not only separated by the high-gain competitive inhibition, but also accumulate over time because of the recurrent amplification, and do so with asymmetric rates as a result of competitive inhibition impinging on the amplifiers. Consequently, the option with the highest norm is predicted to reach the firing threshold of the downstream ‘output’ neurons first, and to trigger the associated percept or action. An additional advantage of this scheme involving temporal primacy is that it precludes the need for careful tuning of the parameters corresponding to competitive inhibition and amplification (as long as they are within a neurally plausible range).

Thus, high-gain competitive inhibition together with downstream recurrent amplification can selectively enhance responses to the option with the highest norm while suppressing the responses to all other options, thereby allowing only the option with the highest norm to drive output.

#### Neural support

Numerous studies across the selection literature have demonstrated that the responses to the winning option are higher (or enhanced), whereas those to the losing options are low (or suppressed), as described in the previous sections (Freedman et al., 2001; Hernández et al., 2002; Romo et al., 2002; Freedman et al., 2003; Romo et al., 2004; Freedman and Assad, 2006; Padoa-Schioppa and Assad, 2006; Cisek and Kalaska, 2010; Mysore et al., 2011; Mysore and Knudsen, 2011a). However, very little is known about the circuit mechanisms underlying unitary output generation.

In the context of spatial attention, the so called ‘visuomotor’ and ‘motor’ neurons in the primate FEF (Bruce and Goldberg, 1985) as well as the SCid (Wurtz and Albano, 1980) are known to exhibit peak activity just before initiation of a saccade towards the motor field of that neuron. Similarly, caudate neurons in the basal ganglia show selective activity to the target of attention even when no overt action was required (Arcizet and Krauzlis, 2018). Additionally, a recent primate study suggests that the difference between the activities of right and left SCs serves as a decision variable, which, when it crosses a threshold, results in unitary action (Herman et al., 2018). This is in line with an earlier model (for perceptual decision-making) in which a multi-area module involving the primate cortex, basal ganglia and the SC was proposed as a means both for detecting threshold crossing (via the cortico-collicular pathway) as well as for threshold setting (via the cortico-caudate pathway) (Lo and Wang, 2006). In all cases, however, the implementational details of the proposed models in neural circuitry are unknown – for instance, the neural circuit mechanisms for comparison across the SCs, the source of recurrent excitation, etc.

Potential support for the specific implementational scheme that we propose above - namely high-gain competitive inhibition and recurrent amplification, comes from the avian midbrain selection network. Neurons in the avian sensorimotor hub, the OT, receive powerful inhibition from GABAergic neurons in the midbrain tegmentum, called Imc (Marín et al., 2007; Mysore and Knudsen, 2013), following a donut-like spatial pattern (Wang et al., 2004; Mahajan and Mysore, 2020). In addition, cholinergic neurons in the nucleus isthmi pars parvocellularis serve as point-to-point recurrent amplifiers of activity in the OT of birds (Marín et al., 2005; Asadollahi and Knudsen, 2016) as well as fish (Henriques et al., 2019). Indeed, because Ipc neurons are powerfully suppressed by the Imc, the circuit supports asymmetric amplification as well (Wang et al., 2004; Asadollahi et al., 2010). Whether and how this Imc-Ipc circuit, which is thought to be conserved across vertebrates (Knudsen, 2018), impacts unitary output generation in behavior is yet to be tested, and similar circuits are yet to be studied in other species/brain areas.

In the context of perceptual decision-making, studies have demonstrated that unitary choice is triggered immediately after neural firing rates of primate LIP reach a fixed value (Gold and Shadlen, 2007; Churchland and Ditterich, 2012; Hanks and Summerfield, 2017). The observation that the responses to the losing options are not zero, but remain graded, has led others to propose that the LIP may be feeding its activity to a downstream area that implements unitary choice (Gold and Shadlen, 2002; Gold and Shadlen, 2007). (Note that, as we point out above, responses to the losing options don’t necessarily need to go to zero, they just need to not exceed the threshold for output.) Although modeling studies have accounted for threshold generation in perceptual decision-making tasks (Lo and Wang, 2006; Wei et al., 2015), an understanding of the actual neural implementation is still lacking.

In value-based decision-making, a model of LIP activity during choice has used an arg-max operation for unitary choice generation, which is consistent with both the WTA as well as the A2T formulations, but the circuit details are as yet unclear (Louie et al., 2011).

In the context of action selection, neurons in the primate premotor and motor cortices encoding for the winning option, but not the losing ones, are shown to be active at the time of choice (Cisek and Kalaska, 2005). A WTA-like model has been proposed, but the neural implementation is as yet unclear. Some support for inhibition as well as recurrent excitation in the context of voluntary movement initiation has been reported in rats (Isomura et al., 2009).

In summary, unitary choice is self-evident in animal behavior and has been demonstrated in the laboratory across selection tasks and species. We have proposed that a general circuit mechanism underlying this computation is competitive inhibition with high gain, coupled with recurrent amplification. There is clear evidence that such a circuit exists in the avian midbrain selection network. However, the link between the circuit architecture and unitary choice in this network, and more generally, the neural circuit mechanisms underlying unitary choice in other brain areas and in the context of other forms of selection, are yet to be uncovered experimentally.

## Feasibility, generality and limitations of the framework

**Figure 9.**
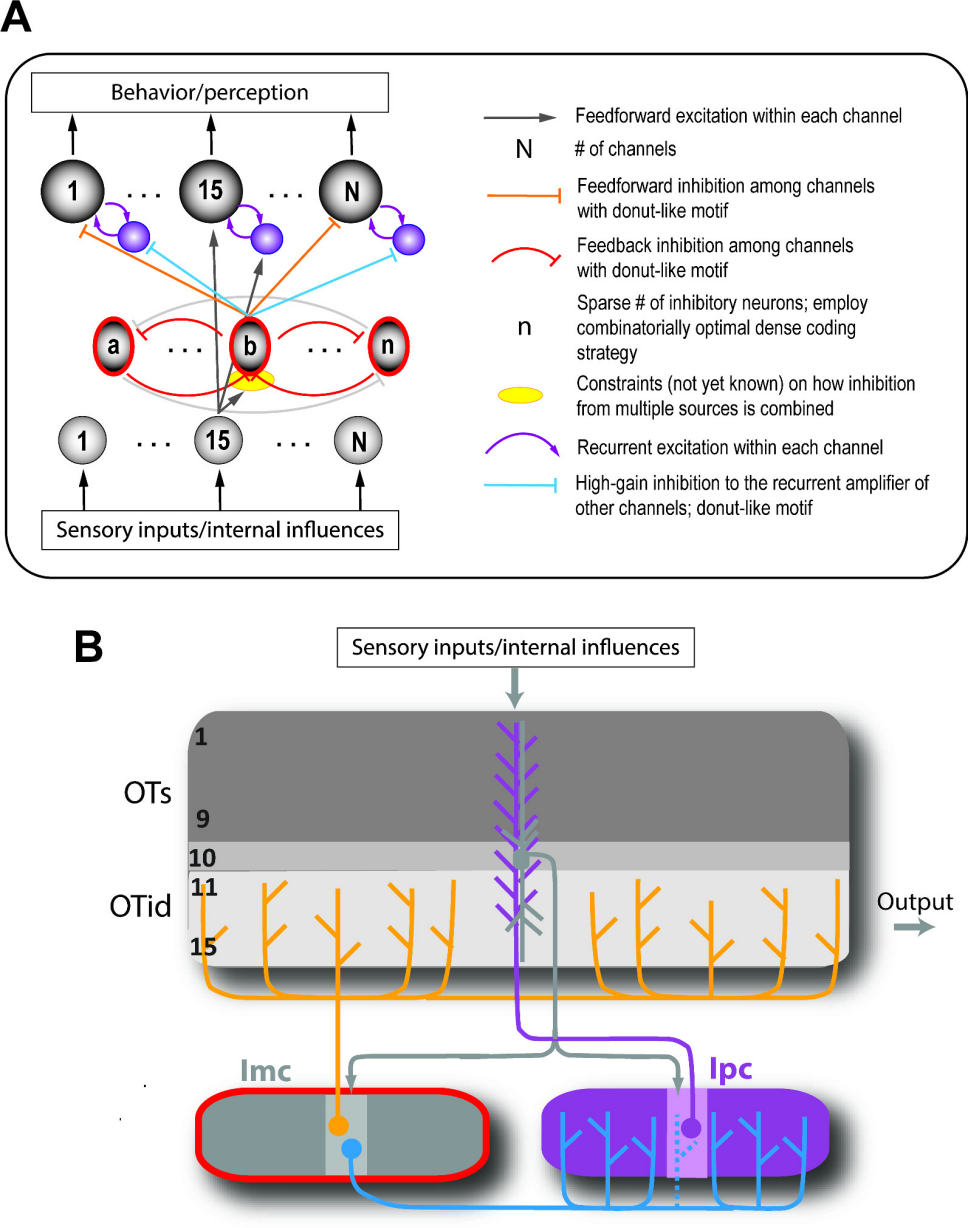
Canonical circuit for competitive selection. (**A**) Schematic combining the circuit motifs from Figure 4 through Figure 8. It represents the proposed neural circuit implementation corresponding to the framework for competitive selection (Figure 1). Only connections with respect to channel 15 are highlighted, for visual clarity. (**B**) The spatial selection circuit in the avian midbrain, thought to be conserved across vertebrates, parallels the proposed circuit. It includes the optic tectum (OT, superior colliculus in mammals), GABAergic isthmi neurons (Imc; grey structure with red outline), and cholinergic isthmi neurons (Ipc; purple structure).

For the purposes of mapping the implementation of competitive selection onto explicit neural circuit elements, we broke it down into a set of six well-defined computational primitives by drawing inspiration from the idealized WTA operation. Starting with a simple neural circuit (Figure 4), we progressively built in these computational features by adding specific motifs to the circuit’s architecture. The resulting combined circuit, capable of implementing all six computational features of WTA selection, is shown in Figure 9A. The routes for circuit implementation proposed here are either the simplest ones and/or are those that are supported directly by published reports. Nonetheless, the specific implementation in use in any particular brain areas and animal species is a question to be answered experimentally.

Notably, although developed to account for the steady state response properties of WTA, this circuit can generate time-evolving responses consistent with the A2T perspective, and can produce predictions related to behavioral reaction times. It is also amenable to the action of a broad urgency signal for multiplicatively modulating sensory evidence, thought to be essential for handling time-varying sensory evidence (Thura and Cisek, 2014). We note that the computations described here are not required to be implemented within a single, monolithic ‘selection circuit’, rather, they may well be implemented in a distributed fashion across brain areas and processing stages.

At first glance, this combined circuit appears complex with several seemingly specialized circuit components. Consequently, it raises two critical questions: (a) First, is it biologically plausible? In other words, is there evidence that such a circuit is implemented (either in a combined or distributed fashion) in the brain in the context of any selection task? (b) Second, is it general? In other words, does it represent a specially curated solution that applies only in limited contexts, or can it generalize for neural implementation across brain areas and animal species? We turn to these questions next.

### Plausibility: Biological instantiation of the combined circuit

Brain networks in several animal species including birds, fish, reptiles (Mysore and Knudsen, 2011b; Jovanic et al., 2016; Koyama et al., 2016; Knudsen, 2018; Koyama and Pujala, 2018; Mahajan and Mysore, 2018; Fernandes et al., 2019; Mahajan and Mysore, 2020) implement most of the six computational features in the manner depicted in the combined circuit framework (Figure 9). We focus here, specifically on the avian midbrain selection network as an example case (Sereno and Ulinski, 1987; Wang et al., 2004; Wang et al., 2006; Knudsen, 2011). This network is thought to be conserved across all vertebrates (Knudsen, 2018) and to serve selection for spatial attention. It consists of the optic tectum (OT, the avian SC), and satellite inhibitory (Imc) and cholinergic (Ipc) neurons in midbrain tegmentum (Figure 9B).

The SCid/OTid, which is required for selection for spatial attention (McPeek and Keller, 2004; Lovejoy and Krauzlis, 2010), encodes sensory stimuli as well as internal influences in a topographically organized map of stimulus priority (Fecteau and Munoz, 2006). The inhibitory Imc neurons deliver feedforward inhibition globally across the OTid space map and are required for comparison among competing options (Marín et al., 2007; Mysore et al., 2010; Mysore and Knudsen, 2013). They are connected in a functionally donut-like fashion with the OTid and Ipc neurons, implementing the categorical selection boundaries observed in the OT (Wang et al., 2004; Mahajan and Mysore, 2020). They inhibit one another, potentially allowing for flexible selection boundaries (Mysore and Knudsen, 2012; Goddard et al., 2014). They have been shown to employ a combinatorially optimized strategy to achieve selection at all pairs of stimulus locations (Mahajan and Mysore, 2018). In addition, Imc neurons, which are associated with each location of the OT space map (Mahajan and Mysore, 2018), are thought to help implement selection among multiple competing stimuli, although respecting as yet unknown additional constraints regarding input integration (Rajagopalan et al., 2018). Finally, their function is complemented by that of the cholinergic amplifiers – Ipc neurons, which connect in a point-to-point, recurrent manner with the OTid (Marín et al., 2005; Wang et al., 2006; Asadollahi and Knudsen, 2016), and are also the recipients of donut-like inhibition from the Imc (Mahajan and Mysore, 2020), potentially facilitating unitary choice.

Thus, there is direct evidence that the avian (as well as fish and reptilian) midbrain network employs the proposed circuit motifs for the first four of the six ‘hidden’ computational features of competitive selection (Figure 1, Box 1), with the ones underlying the other two - selection amidst clutter and unitary choice, remaining to be investigated.

In addition, in several other species, there is evidence for the plausibility of the circuit implementation needed for a subset of the computational features. For instance, (a) long-range inhibition in monkey cortex (Tamamaki and Tomioka, 2010; Womelsdorf and Everling, 2015), monkey LIP (Falkner et al., 2010), rodent hippocampus/entorhinal cortex (Picardo et al., 2011), and cross-hemispherical suppress (Sooksawate et al., 2011); (b) donut-like inhibition in flies (Jovanic et al., 2016), fish (Koyama et al., 2016; Koyama and Pujala, 2018), and rodent amydgala (Fadok et al., 2017); (b) reciprocal inhibition of inhibition in flies (Jovanic et al., 2016), fish (Koyama et al., 2016; Koyama and Pujala, 2018), and rodent amydgala (Fadok et al., 2017).

### Generality: Comparison with leading computational models of competitive selection

To address the issue of generality, we next compare our framework with computational models that have been proposed in the literature to account for behavioral and neural responses (across brain areas) in a range of selection and decision-making tasks.

Models of competitive selection fall into two broad classes based on the nature of their architectures – those that involve structured connectivity diagrams (‘structured’ models), and those that are highly recurrent with no clear apriori structure imposed on them (Mante et al., 2013; Chaisangmongkon et al., 2017). The latter can account for mixed selectivity of neural responses observed in cortical areas (but see Hirokawa et al., 2019, and can be trained to learn different category boundaries. However, it is unclear whether they are dynamically flexible – i.e., able to adjust category boundaries on the fly, especially without the use of indicator or gating variables that convey discrete group information (Siu et al., 1991). Importantly, because it is difficult to extract specific circuit mechanisms from the latter, we do not consider them further. We compare our circuit framework with the former class of ‘structured’ models of competitive selection.

**Figure 10.**
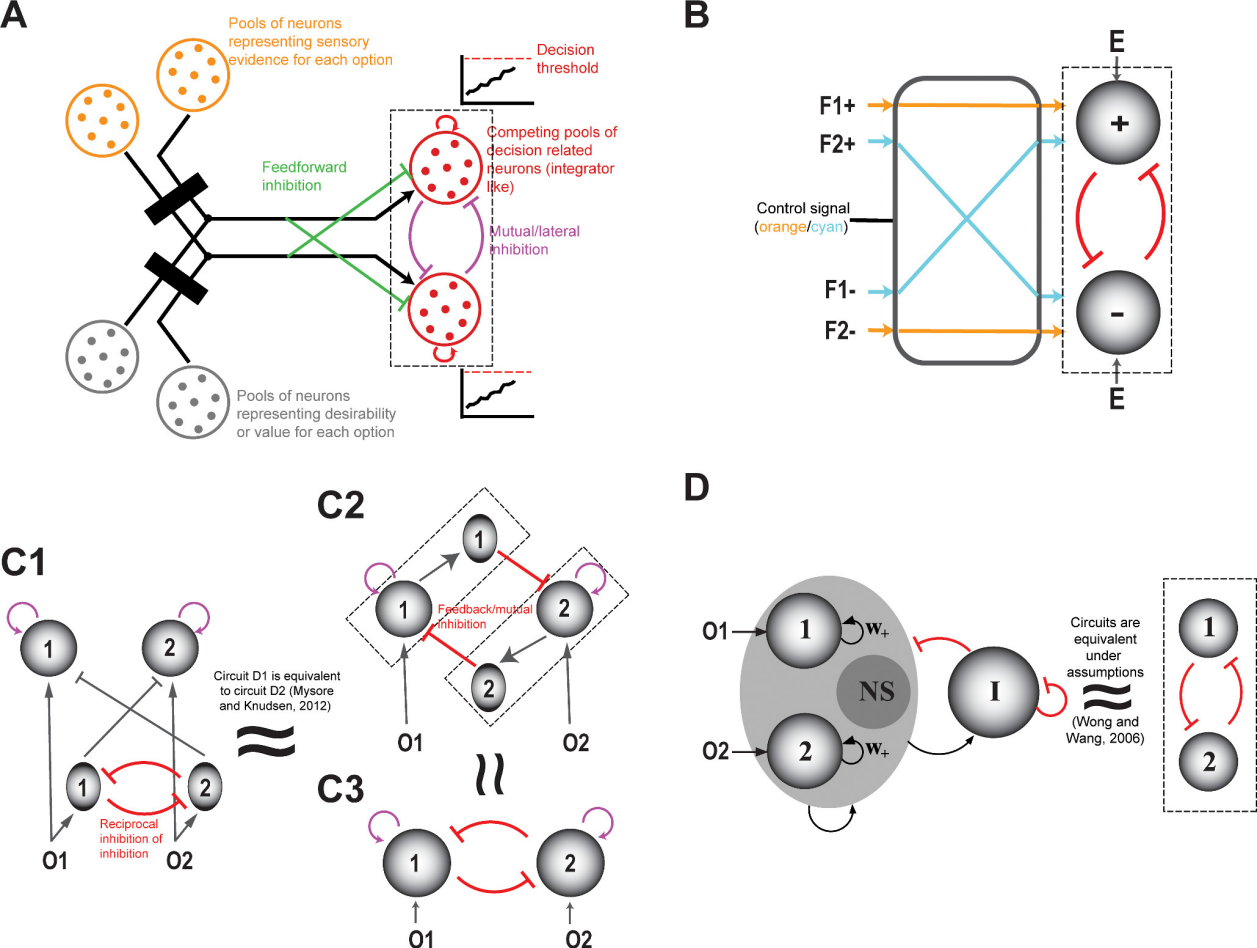
Comparison with prominent models of competitive selection. In all panels, shaded circles and ovals represent neurons or neural populations; arrows with triangular heads indicate excitatory connections, and with flat heads, inhibitory connections. O1 and O2 represent two options. (**A**) Schematic of a model of decision-making adapted from Roe et al., 2001, Usher and McClelland, 2001; Usher and McClelland, 2004, Churchland and Ditterich, 2012. (**B**) Model of decision-making and working memory in a tactile delayed match-to-sample task adapted from Machens et al., 2005. F1 is the first stimulus presented (tactile stimulation), F2 is the stimulus presented after a delay. F1+/F2+ (F1-/F2-) represent neurons that respond with increasing (decreasing) firing rates for increasing tactile stimulation frequency. The (+) and (-) represent pools of decision making neurons which mutually inhibit each other via feedback inhibition. The control signal helps route information from the F+/F- pools of neurons to the decision-making pools. The two states of the control signal are depicted here with two colors – orange and cyan. (**C**) C1 shows a 2-option version of the competitive selection framework proposed here (and found in several species – see subsection titled ‘Plausibility: Biological…’). C2 shows a circuit equivalent of the model in C1 (Mysore and Knudsen, 2012) which can be reduced to the classic mutual inhibition model. C3 shows the high-level, mutual inhibition model that summarizes all the models discussed in A,B and C; same as Figure 2C. Note that C2 implements a combination of feedback inhibition and donut-like inhibition (Mahajan and Mysore, 2020). (**D**) A recurrent network model of decision-making and time-integration (Amit and Brunel, 1997; Wang, 2002; Lo and Wang, 2006; Wong and Wang, 2006; Furman and Wang, 2008; Louie et al., 2011; Deco et al., 2013) (Adapted from Wong and Wang, 2006). Under special assumptions of linear input-output functions and gating variables, this model also reduces to the classic mutual inhibition model in C3. I: denotes population of inhibitory neurons; NS: denotes population of neurons nonselective for either input.

Structured models can themselves be divided into two kinds. First, models in which the inhibition is structured (Figure 10A–C; Roe et al., 2001; Usher and McClelland, 2001; Usher and McClelland, 2004; Machens et al., 2005; Bogacz et al., 2007; Churchland and Ditterich, 2012; Mysore and Knudsen, 2012), and second, models in which the inhibition is unstructured (pooled; Figure 10D **-**left panel, Amit and Brunel, 1997; Wang, 2002; Lo and Wang, 2006; Wong and Wang, 2006; Furman and Wang, 2008). The structured inhibition models include models of multialternative decision-making (Figure 10A, Roe et al., 2001; Usher and McClelland, 2004; Bogacz et al., 2007; Churchland and Ditterich, 2012), of perceptual decision-making (Figure 10B, Machens et al., 2005), and of selection for spatial attention (Mysore and Knudsen, 2012). Notably, there is a great deal of commonality between these models and our framework (Figure 10C), with all of them being elaborations of a core conceptual circuit diagram involving mutually inhibiting populations of neurons (Figure 10C3). The unstructured inhibition models, which include models of perceptual decision-making (Amit and Brunel, 1997; Wang, 2002; Lo and Wang, 2006; Wong and Wang, 2006; Furman and Wang, 2008) and value-based decision-making (Louie et al., 2011; Deco et al., 2013), stand as a potential alternative to our framework. However, such circuits involving pooled uniform inhibition may be less effective at generating categorical (step-like) neural selection boundaries (Carandini and Churchland, 2013; Mahajan and Mysore, 2020), and may also not account fully for decision behavior among multiple alternatives (Gluth et al., 2020). Incidentally, it has been shown that under special circumstances, these pooled models can reduce to the same mutually inhibitory module (Figure 10D- right panel (Wong and Wang, 2006). Below, we compare and contrast these leading structured models with each of the key components of our circuit framework.

In the context of building the representational landscape for competing options, we proposed that the norm of each option constitutes a means for lawfully comparing options. A straightforward perspective is that the norm represents the net worth of an option, such that the contribution of each attribute is combined in some weighted fashion to produce a single number. However, this is not necessary. *Alternatives:* In the case of hierarchical or distributed implementation of selection, subsets of attributes may be progressively represented along the stages of a hierarchy (Cisek, 2012; Connor and Stuphorn, 2015; Lorteije et al., 2015; Hunt and Hayden, 2017), or alternatively, intra-attribute competition could occur sequentially (Roe et al., 2001; Usher and McClelland, 2004; Churchland and Ditterich, 2012; Chen et al., 2013). Indeed, subsets of attributes could themselves determine entirely the final choice (Farashahi et al., 2019; Rouault et al., 2019). In these cases, the norm would correspond not to the ‘net worth’ of each option, but just to the ‘worth’ of the relevant attribute(s). Nonetheless, the core idea is that options, or subsets of their attributes, are being competed along some common axis, with the competition implemented by circuit elements in Figure 9A.

In the context of the first ‘hidden’ computational feature, our ‘baseline’ circuit established a means for the neural representations of competing options to be compared, and did so with feedforward inhibition distributed from each option to all. *Comparison:* This motif is found in several of the leading computational models (Bogacz and Gurney, 2007; Olsen et al., 2010; Figure 10A). *Alternative*: Whereas inhibitory interactions between competing options are important for comparing among them, the inhibition does not need to be of the feedforward variety, even though such inhibition is found in many brain areas. Feedback inhibition, a mainstay of many selection models (Roe et al., 2001; Usher and McClelland, 2001; Machens et al., 2005; Bogacz and Gurney, 2007; Ratcliff and McKoon, 2008; Churchland and Ditterich, 2012; Mysore and Knudsen, 2012), can also serve the purpose of comparing among options (Mahajan and Mysore, 2020), and is consistent with our circuit framework (see the paragraph after next).

In the context of the second ‘hidden’ computational feature, we introduced a donut-like inhibitory motif for generating categorical (step-like) neural response profiles (Mahajan and Mysore, 2020). *Comparison:* The donut-like inhibitory motif is found in all of the leading structured inhibition models (Roe et al., 2001; Usher and McClelland, 2001; Machens et al., 2005; Bogacz and Gurney, 2007; Ratcliff and McKoon, 2008; Churchland and Ditterich, 2012; Mysore and Knudsen, 2012). Unstructured inhibition models of decision-making, which invoke pooled uniform inhibition, do not possess the donut-like motif (Amit and Brunel, 1997; Wang and Spelke, 2002; Lo and Wang, 2006; Wong and Wang, 2006; Furman and Wang, 2008; Louie et al., 2011; Deco et al., 2013), but such circuits may not be effective for generating categorical neural selection boundaries (Carandini and Heeger, 2012; Mahajan and Mysore, 2020). *Alternative*: The donut-like motif for categorization can be implemented not only via the feedforward inhibitory path among the options, but also via a feedback path between the options, or via both (Mahajan and Mysore, 2020). Indeed, it is implemented in the feedforward as well as feedback inhibitory pathways in most of the leading structured inhibition models (Roe et al., 2001; Usher and McClelland, 2001; Bogacz and Gurney, 2007; Ratcliff and McKoon, 2008; Churchland and Ditterich, 2012; Mysore and Knudsen, 2012), and in just the feedback pathway in others (Machens et al., 2005).

In the context of the third ‘hidden’ computational feature, we added feedback inhibition between the competing options to implement flexible selection boundaries (Mysore and Knudsen, 2012). *Comparison:* Feedback inhibition is, in fact, found in nearly all the leading models with structured inhibition (Roe et al., 2001; Usher and McClelland, 2001; Machens et al., 2005; Churchland and Ditterich, 2012). Models with unstructured inhibition have been shown to display flexibility (Carandini et al., 1997), though the reason for this is unclear. It has been proposed that inhibition among the pool of inhibitory neurons found in these models may be the key (Mysore and Knudsen, 2012), but this is yet to be tested. *Alternative:* Both *direct* reciprocal inhibition among inhibitory neurons, as well as *indirect* feedback inhibition routed through intermediate excitatory neurons, can achieve flexibility (Mysore and Knudsen, 2012). In the owl midbrain selection circuit, feedback inhibition is implemented in the direct manner (Goddard et al., 2014). In the structured inhibition models listed above, feedback inhibition is typically implemented in an indirect manner.

In the context of the fourth ‘hidden’ computational feature, we examined the problem of selection among many (all) viable pairs of options. Based on a recent study of spatial selection (Mahajan and Mysore, 2018), we incorporated a combinatorial solution into the circuit framework as a biologically efficient solution. *Comparison*: To the best of our knowledge, the mechanistic underpinnings of this computational feature have not been investigated in other studies. *Alternative*: A potentially straightforward alternative to the combinatorially optimized solution is a simple ‘copy-and-paste’ strategy, in which the RFs (or preference tuning curves) of the inhibitory neurons closely match those of the input excitatory neurons (Mahajan and Mysore, 2018). However, this solution is metabolically expensive (Mahajan and Mysore, 2018), and therefore, less desirable. Another potential alternative may be the (currently unknown) mechanism underlying value 'remapping' reported recently (Xie and Padoa-Schioppa, 2016).

In the context of the fifth ‘hidden’ computational feature, we considered the problem of selection among multiple (>2) options. Based on preliminary data (Rajagopalan et al., 2018), we highlighted the potential importance of nonlinear combination of inputs in order to account for the effects of multiple options on flexibility of the selection boundary, an aspect of multi-option decision-making that has not been experimentally investigated in other studies. *Comparison:* Broadly, existing models involving structured inhibition achieve selection among multiple options by essentially scaling-up the circuit diagram for two options to multiple ones; ours does this as well. Additionally, one set of models (Bogacz and Gurney, 2007; Bogacz et al., 2007) specifically calls for nonlinear combination of inputs, consistent with our proposal. *Alternative*: Other structured inhibition models use linear or nonlinear integrators, allow negative activity, and/or employ constant versus variable feedback activation to account for multi-option selection (Ditterich, 2010; Churchland and Ditterich, 2012). However, it remains to be explored whether they are able to also account for the effects on flexibility that non-linearity does. Regarding models with unstructured inhibition (Furman and Wang, 2008), recent evidence suggests that they may not account fully for decision behavior among multiple alternatives (Gluth et al., 2020).

Finally, in the context of the sixth ‘hidden’ computational feature, we proposed a ‘push-pull’ solution for the unitary choice problem, involving an amplifier downstream of the inhibitory neurons. *Comparison/Alternative*: To the best of our knowledge, explicit biological solutions for solving the unitary choice problem are not known. Existing computational models typically state that a threshold must be crossed for the decision, but do not provide a neural mechanism for it (Churchland and Ditterich, 2012; Huk and Meister, 2012).

Taken together, there are many points of correspondence between our proposed circuit framework in Figure 9A, and computational models with structured inhibition that have been proposed to account for selection behaviors (Figure 10A–C). There is also little apparent difference between them in terms of overall complexity (Figure 9A vs. Figure 10A–C). Our framework, in essence, serves as a collection of mechanistic building blocks underlying many of the models of selection and decision-making. This point is further illustrated in Figure 10C: abstracting away the implementational details of our combined circuit reduces it to a circuit diagram involving mutually inhibiting populations of neurons, which is identical to the one used to represent each of the leading models with structured inhibition.

### A canonical neural circuit framework

The construction of our framework (Figure 9A) from first principles, by using a computational primitive-centric approach, has resulted in potential insights into the complex function of competitive selection. It allows for the deconstruction of a potential selection circuit into core motifs that are associated with specific computations, thereby providing a starting point for the experimental search for neural circuit mechanisms of competitive selection (see section titled ‘Experimentally testable predictions’). By contrast, whereas each of the existing computational models successfully accounts for data from the selection task(s) that it is built to model, it is not always clear why that model works, what circuit elements are essential, and what experiments to design in order to investigate the neural circuit underpinnings of selection in a relevant brain area. Indeed, in some cases, intuition regarding the computational roles of different circuit elements does not appear to be borne out: for instance, it has recently been demonstrated that recurrent amplification as well as feedback inhibition, by themselves, are insufficient to generate categorical selection boundaries (Mahajan and Mysore, 2020), in contrast to previous conjectures.

In light of the links to biology and parallels to existing models, the circuit framework proposed here is potentially capable of accounting for the implementation of selection in different tasks and across different brain areas. This will likely involve the use of alternate, but equivalent, implementations of the proposed core circuit motifs as discussed above, and may involve a distributed implementation of the various computational features across processing stages rather than a combined implementation within one so-called selection circuit. We propose that the building blocks we identify here may be mixed and matched, based on the needs and flavor of a particular selection task, to build a neural circuit to solve it.

### Limitations and caveats

An important part of our framework is the representational landscape in which competing options, or more generally, the attributes that are being compared, are encoded along some common scale of comparison (norm), and in some common currency. We pointed to firing rate as a potential common currency, and firing rate-dependent inhibition featured prominently in the remainder of the framework. However, it is unclear if firing rate is the only such currency, and if not, the manner in which the subsequent computations are to be implemented is not clear in the current framework. Related to this point, the framework is also agnostic to how the norm comes to be encoded (see also Box 4), especially considering recent reports that the norm is not universal and can depend on the context (Farashahi et al., 2019; Rouault et al., 2019; Koechlin, 2020).

Second, although we discuss six computations in the context of WTA symmetry-breaking, it is possible that they are not all equally important, in general. Comparison and unitary choice, the first and last ‘hidden’ computational features, are likely universal, with the others being useful depending on the nature of the selection task and the animal’s behavioral goals. For instance, categorical neural selection boundaries, though common, may not always be necessary. Similarly, not all selection tasks may require flexibility, with fixed boundaries being not only sufficient, but also necessary in some cases. For instance, in the case of syllable-length identification by songbirds, the goal of the underlying neural circuit is the determination of how the length of a syllable compares to a fixed value (Prather et al., 2008). With respect to the ability to select among all viable options, combinatorially optimized inhibition implemented by sparse inhibitory neurons with dense coding properties has been demonstrated as an efficient solution for selection across spatial locations (see 3). However, it is unclear if this is the only solution, especially in the context of other forms of selection (Xie and Padoa-Schioppa, 2016). Finally, for selection among more than two options, much is still unknown about specifics of neural circuit implementation.

Third, our framework was constructed around the WTA operation. Although it admits the analysis of response timecourses, choice response times were not considered explicitly.

Despite these limitations, the explicit mapping in our framework, of computations for selection onto circuit elements, can serve as a useful starting point for the investigation of circuit mechanisms underlying different forms of selection. Specific, experimentally testable predictions of the framework are outlined, next.

## Experimentally testable predictions

### Comparison: source of inhibition

#### Predictions

(a) Neural representations of options are modulated (suppressed) in the presence of competing ones, and (b) neural inhibition plays a key role, and it operates globally in the space of options.

#### Experimental tests

(a) Compare neural responses (in the relevant brain area) to one option versus two competing options and evaluate if response reduction occurs. (b1) Characterize anatomically the underlying neural circuit in detail and assess if inhibitory neurons are part of the circuit (local or distant). (b2) If so, test if disrupting neural inhibition minimizes/abolishes the impact of one option on another; test also the scale or scope of this inhibition with respect to the space of options by systematically changing one the competing options.

#### Significance

These experiments can identify the potential source of inhibition for implementing comparisons among options, reveal if it is of the structured variety or the unstructured (pooled) variety, and whether inhibition is implemented via long-range inhibitory projections or long-range excitatory projections onto local inhibitory neurons.

### Categorical selection boundary

#### Predictions

(a) Neural representations underlying selection/decision-making are explicitly categorical. (b) An underlying donut-like pattern of competitive inhibition controls these categorical selection boundaries (Mahajan and Mysore, 2020). (c) Disrupting the categorical nature of the neural representation (by causally perturbing the underlying mechanism) causes a degradation of selection behavior specifically around the category boundary.

#### Experimental tests

(a) Measure responses of a neuron that encodes for (prefers) option A, while also presenting a competing option B. Systematically increase norm (priority, subjective value, etc) of option B from values lower than the norm of option A to values higher than that of A (Mysore et al., 2011; You and Mysore, 2020). The resulting response curve, called a CRP (competitor-norm dependent response profile), is expected to show a reduction in responses with increasing norm of option B. Characterize the categorization index of this CRP in a manner that takes into account response variability, and evaluate if it is significantly greater than 0 (Mahajan and Mysore, 2020). (b1) Based on the anatomical characterization above, determine if a donut-like motif is operational in the circuit. (b2) If so, selectively introduce self-inhibition into the circuit (i.e., disrupt the donut-like motif), repeat the CRP measurement, and re-compute the categorization index (Mahajan and Mysore, 2020). It is expected to be substantially lower than before (and potentially not distinguishable from 0). In other words, the responses are expected to be far less categorical without the donut-like motif. (c) Upon introducing self-inhibition (per b2, above), measure also the effect on behavioral responses and compare them to responses in the intact condition, specifically for stimulus options that straddle the selection boundary.

#### Significance

These experiments can reveal if neural responses (and behavior) underlying the selection task are categorical, if there is a donut-like organization of inhibition, if this is implemented via a feedforward or a recurrent path, and whether it controls categorization.

### Flexibility of selection boundary

#### Predictions

(a) The selection boundary is dynamically flexible, and (b) feedback inhibition between competing options controls flexibility.

#### Experimental tests

(a) Measure CRP at a neuron that encodes for (prefers) option A while also presenting a competing (non-preferred) option B. Repeat this measurement in an interleaved manner with one exception: increase the norm of option A to a different (higher value). For each CRP, determine the norm of option B (‘transition’ norm) at which responses drop from a high value to a low value. If this transition norm is coupled to, and shifts with, the strength of option A, the selection boundary is dynamically flexible (no training/plasticity involved; Mysore et al., 2011). (b1) With anatomical (as well as functional) characterization of the circuit, assess if feedback inhibition exists between neurons encoding different options. Examine if this feedback pathway is direct (reciprocal inhibition among inhibitory neurons) or indirect (routed through intermediate excitatory neurons; Mysore and Knudsen, 2012). (b2) Selectively silence feedback inhibition between options (while leaving the inhibitory neurons themselves intact), and repeat measurements of the two response curves in (a). Depending on how the circuit is organized, this may be achieved either by silencing the feedback projections from one channel onto the other (source-side manipulation), or by preventing neurons in one channel from receiving (feedback) inhibition from those of another (recipient-side manipulation). The manipulation is expected to abolish the shifting of the boundary (transition norm) between the two response curves (Mysore and Knudsen, 2012), while leaving intact the response reduction in each curve.

#### Significance

These experiments can reveal dynamically flexible selection boundaries, the presence of direct vs. indirect feedback inhibition between competing options, and also the circuit mechanism underlying flexibility.

### Ability to select among many (all) viable pairs of competing options

#### Predictions

(a) Animals are able to select between many different pairs of competing options. (b) This ability is mediated by an overcomplete set of inhibitory neurons with sparse coding, or by a sparse set of inhibitory neurons with combinatorially-optimized dense coding of option space (Mahajan and Mysore, 2018), or by an as yet unknown mechanism that results in norm-remapping (Xie and Padoa-Schioppa, 2016). Below we will discuss experiments to test the first two possibilities.

#### Experimental tests

(a) Present animals with different pairs of options (the laboratory equivalent of having to select between apples and oranges, bananas and blueberries, etc). Animals are expected to be able to perform well and consistently. (b1) Within the anatomically identified network relevant to this behavior, identify neurons that drive the inhibition: these can be inhibitory neurons that directly deliver competitive inhibition, or excitatory neurons with long-range connections that deliver inhibition indirectly by driving local inhibitory neurons that largely serve as simple sign-changers (Schryver et al., 2020). Measure responses of these ‘driver’ neurons to various options (individually), and characterize their encoding preferences. Dense coding by these neurons is indicative of a combinatorially-optimized solution, which can be tested with additional analysis/experiments (Mahajan and Mysore, 2018; Mahajan and Mysore, 2020), whereas sparse/ordered coding by these neurons is indicative of a copy-and-paste solution. (b2) ‘Focal’ or selective disruption of a subset of the driver neurons should affect selection between some pairs of options (all the ones that activate these neurons), but not other option pairs.

#### Significance

Results can reveal the neural circuit mechanisms that implement the ability to select among many (all) different pairs of options.

### Ability to select among multiple (>2) competing options

#### Predictions

(a) Animals are able to select among multiple options in a manner that generalizes from selection between two options, but with potential preference reversal effects and contextual effects, and potential shifts in the encoding of the category boundary by individual neurons (Rajagopalan et al., 2018). (b) The neural and behavioral results are accounted for by circuits that involve non-linear combination of inhibitory inputs (Bogacz and Gurney, 2007; Rajagopalan et al., 2018). (c) Disruption of the nonlinearity in the combination of multiple inhibitory inputs impacts selection among multiple (but not two) options.

#### Experimental tests

(a) Present animals with two vs. more than two options, in each case using a CRP stimulus protocol that systematically varies relative norm between the competing options. Measure behavior as well as neural responses and quantify the effect of increasing the number of options on behavioral performance as well as on the neural specification of the selection boundary (i.e., the transition norm; Asadollahi et al., 2011; Mysore and Knudsen, 2011a; Schryver et al., 2020; You and Mysore, 2020). (b) Compare observed outcomes with those predicted by computational models with linear vs. non-linear combination of inhibition, in order to identify the best account for the observed data.

#### Significance

Results will shed light on the neural implementation of a circuit for selection among multiple options, and reveal rules (nonlinearities) governing the integration of inputs from multiple options for such selection.

### Producing a unitary choice

#### Predictions

High gain competitive inhibition coupled (downstream) with high-gain amplification is involved in the production of unitary choice. Specifically, disruption of high-gain amplification by *enhancing* amplification of the option that would normally lose, increases the likelihood of that option being selected, but will slow reaction times, consistent with a change in the decision threshold.

#### Experimental tests

(a) Within the anatomically identified network relevant to the selection behavior, investigate the presence and connectivity of amplifier neurons (cholinergic or glutamatergic, for instance). (b) Assay the strength (gain) of amplification by comparing neural responses to a single option without and with silencing of the ‘amplifier’ neurons (Marín et al., 2005; Asadollahi and Knudsen, 2016). (c) During a two option selection task, experimentally disrupt the high-gain amplification by enhancing the output of the amplifier neurons to the weaker option, and measure neural and behavioral outcomes.

#### Significance

These experiments can reveal the neural circuit mechanism involved in setting the ‘threshold’ for selection and the production of unitary choice.

Box 4. Open questions and challenges.

1. Representation of options and their norms.
  - What is the norm of each option? Is it unidimensional or can it be reduced to a unidimensional value? What is its common currency?
  - How is each option represented – ordered (topographic) or non-ordered and distributed over neural ensembles?
  - How are downstream neural circuits organized to decode this (potentially complex) representation and extract useful quantities necessary to implement the ‘hidden’ computations for selection?
  - What is the source of the motor urgency signal and how it is incorporated into the representational landscape?
2. Properties and organization of inhibition, and recurrent amplification. 
  - Is feedforward neural inhibition implemented in the circuit?
  - If so, how – via long-range inhibitory projections (Tamamaki and Tomioka, 2010; Womelsdorf et al., 2014; Womelsdorf and Everling, 2015) or via long range excitatory projections that impinge on to local inhibitory neurons (Zhang et al., 2014). 
  - Does feedback inhibition exist and if so what is its organization? 
  - What are the properties of inhibitory neurons (preferences, input/output functions) and what rules govern the combination of inhibition from multiple sources. 
  - What is the source of recurrent amplification? How does the competitive inhibition in the circuit interact with the mechanisms of amplification?
3. Unitary choice generation. 
  In which brain area should the implementation of unitary choice be investigated? Only the brain area that occupies the very last computational stage before output generation? This is important to determine because explicitly categorical representations have been reported at various stages all along the neural information processing stream: from areas close to the sensory periphery (Niessing and Friedrich, 2010; Asadollahi et al., 2011; Schryver and Mysore, 2019), to higher order, integrative areas (Freedman et al., 2001; Freedman et al., 2003; Freedman and Assad, 2006; Swaminathan and Freedman, 2012), to areas close to motor output production (Mysore et al., 2011).
4. Integration across multiple brain areas. 
  How do the multiple areas that are typically involved in a particular type of competitive selection task operate synergistically to drive the behavioral or perceptual output of that selection?
5. Overlapping or multiplexed neural function. 
  How to understand, as a whole, the role of neurons in a particular brain area, when that area/neurons are involved in multiple forms of selection?
6. Comparison across species. 
  - What computational and implementational principles of selection are conserved across species? 
  - What principles are specialized?
